# How recombinant swollenin from *Kluyveromyces lactis *affects cellulosic substrates and accelerates their hydrolysis

**DOI:** 10.1186/1754-6834-4-33

**Published:** 2011-09-23

**Authors:** Gernot Jäger, Michele Girfoglio, Florian Dollo, Roberto Rinaldi, Hans Bongard, Ulrich Commandeur, Rainer Fischer, Antje C Spiess, Jochen Büchs

**Affiliations:** 1 AVT-Aachener Verfahrenstechnik, Biochemical Engineering, RWTH Aachen University, Worringerweg 1, D-52074 Aachen, Germany; 2 Institute of Molecular Biotechnology, RWTH Aachen University, Worringerweg 1, D-52074 Aachen, Germany; 3 Max-Planck-Institut für Kohlenforschung, Kaiser-Wilhelm-Platz 1, D-45470 Mülheim an der Ruhr, Germany; 4 Fraunhofer Institute for Molecular Biology and Applied Ecology (IME), Forckenbeckstrasse 6, D-52074 Aachen, Germany; 5 AVT-Aachener Verfahrenstechnik, Enzyme Process Technology, RWTH Aachen University, Worringerweg 1, D-52074 Aachen, Germany

## Abstract

**Background:**

In order to generate biofuels, insoluble cellulosic substrates are pretreated and subsequently hydrolyzed with cellulases. One way to pretreat cellulose in a safe and environmentally friendly manner is to apply, under mild conditions, non-hydrolyzing proteins such as swollenin - naturally produced in low yields by the fungus *Trichoderma reesei*. To yield sufficient swollenin for industrial applications, the first aim of this study is to present a new way of producing recombinant swollenin. The main objective is to show how swollenin quantitatively affects relevant physical properties of cellulosic substrates and how it affects subsequent hydrolysis.

**Results:**

After expression in the yeast *Kluyveromyces lactis*, the resulting swollenin was purified. The adsorption parameters of the recombinant swollenin onto cellulose were quantified for the first time and were comparable to those of individual cellulases from *T. reesei*. Four different insoluble cellulosic substrates were then pretreated with swollenin. At first, it could be qualitatively shown by macroscopic evaluation and microscopy that swollenin caused deagglomeration of bigger cellulose agglomerates as well as dispersion of cellulose microfibrils (amorphogenesis). Afterwards, the effects of swollenin on cellulose particle size, maximum cellulase adsorption and cellulose crystallinity were quantified. The pretreatment with swollenin resulted in a significant decrease in particle size of the cellulosic substrates as well as in their crystallinity, thereby substantially increasing maximum cellulase adsorption onto these substrates. Subsequently, the pretreated cellulosic substrates were hydrolyzed with cellulases. Here, pretreatment of cellulosic substrates with swollenin, even in non-saturating concentrations, significantly accelerated the hydrolysis. By correlating particle size and crystallinity of the cellulosic substrates with initial hydrolysis rates, it could be shown that the swollenin-induced reduction in particle size and crystallinity resulted in high cellulose hydrolysis rates.

**Conclusions:**

Recombinant swollenin can be easily produced with the robust yeast *K. lactis*. Moreover, swollenin induces deagglomeration of cellulose agglomerates as well as amorphogenesis (decrystallization). For the first time, this study quantifies and elucidates in detail how swollenin affects different cellulosic substrates and their hydrolysis.

## Background

Naturally occurring lignocellulose is a promising starting material for the sustainable production of platform chemicals and fuels [1-6]. The hydrolysis of its main component cellulose to glucose necessitates a cellulase system consisting of cellobiohydrolase (CBH, E.C. 3.2.1.91), endoglucanase (EG, E.C. 3.2.1.4) and β-glucosidase (E.C. 3.2.1.21) [7-9]. Besides enzyme-related factors (for example, enzyme inactivation and product inhibition) [10], the enzymatic hydrolysis of cellulose is limited by its physical properties [11-14]. These properties, in particular, are the degree of polymerization, accessibility and crystallinity [15-18]. Cellulose accessibility, which is determined by cellulose particle size (external surface area) and porosity (internal surface area) [15,19], is the most important factor for hydrolysis [15,18,20-24]. This accessibility reflects the total surface area available for direct physical contact between cellulase and cellulose and, therefore, influences cellulase adsorption as well as the rate and extent of cellulose hydrolysis [21,25]. Furthermore, crystallinity is a relevant factor for cellulose hydrolysis, since it influences the reactivity of adsorbed cellulases [26]. Here, it should be noted that crystallinity may also affect cellulase adsorption [26,27] and, therefore, cellulose accessibility [15,21,28]. Up to now, the relationship between crystallinity and accessibility has not been clearly understood [15,29]. However, for high cellulose hydrolysis rates and yields, cellulose accessibility needs to be increased and, conversely, its crystallinity reduced [30,31]. To achieve this and accordingly improve subsequent hydrolysis, pretreatment techniques are essential [6,14,16,32].

Since pretreatment can be expensive, there is a prime motivation to screen and improve it [33-37]. Over time, many pretreatment technologies have been developed: physical (for example, milling or grinding), physicochemical (for example, steam explosion or ammonia fiber explosion), chemical (for example, acid or alkaline hydrolysis, organic solvents or ionic liquids), biological or electrical methods, or combinations of these methods [33,35]. Some of these techniques entail expensive equipment, harsh conditions and high energy input [33]. By contrast, in the past years, non-hydrolyzing proteins have been investigated that pretreat cellulose under mild conditions [17,20]. After regular lignocellulose pretreatment, these non-hydrolyzing proteins can be added during cellulose hydrolysis [38] or they can be utilized in a second pretreatment step in which cellulose is the substrate [17].

During this second pretreatment step, cellulose is incubated under mild conditions with non-hydrolyzing proteins that bind to the cellulose. As a result, cellulose microfibrils (diameter around 10 nm [39,40]) are dispersed and the thicker cellulose macrofibrils or fibers (diameter around 0.5 to 10 μm, consisting of microfibrils [39-41]) swell, thereby decreasing crystallinity and increasing accessibility [20,42-44]. This phenomenon was named amorphogenesis [20,42]. Furthermore, cellulose-binding proteins can lead to deagglomeration of cellulose agglomerates (diameter > 0.1 mm, consisting of cellulose fibers) [45,46], thereby separating cellulose fibers from each other and additionally increasing cellulose accessibility. Ultimately, amorphogenesis as well as deagglomeration promote cellulose hydrolysis [20].

Various authors have described hydrolysis-promoting effects when pretreating cellulose with single cellulose-binding domains [17], expansins from plants [38,47-49] or expansin-related proteins from *Trichoderma reesei *[50], *Bacillus subtilis *[51], *Bjerkandera adusta *[52] or *Aspergillus fumigatus *[46]. A prominent expansin-related protein is swollenin from the fungus *T. reesei*. In contrast to cellulases, the expression levels of swollenin in *T. reesei *are relatively low (1 mg/L) [50]. Thus, swollenin from *T. reesei *has been heterologously expressed in *Saccharomyces cerevisiae *[50], *Aspergillus niger *[50] and *Aspergillus oryzae *[53]. The expression levels in *S. cerevisiae*, however, are also low (25 μg/L) [50] and only *A. oryzae *produces swollenin in higher concentrations (50 mg/L) [53]. According to Saloheimo *et al*. [50], swollenin can disrupt the structure of cotton fiber or the cell wall of the algae *Valonia macrophysa*. Since swollenin shows a high sequence similarity to plant expansins [50], it may have a similar function and lead to the disruption of cellulosic networks within plant cell walls [20]. Thus, swollenin may have an important role in the enzymatic degradation of lignocellulose by *T. reesei *[54]. Up to now, however, there is no systematic and quantitative analysis of the effects of swollenin on cellulosic substrates and their hydrolysis.

First, this study presents an alternative way of producing recombinant swollenin in order to generate sufficient swollenin for industrial applications. Second, the main objective is to show how recombinant swollenin quantitatively affects relevant physical properties of cellulosic substrates and how it affects their subsequent hydrolysis.

## Results and discussion

### Production and analysis of recombinant swollenin

**Figure 1:**
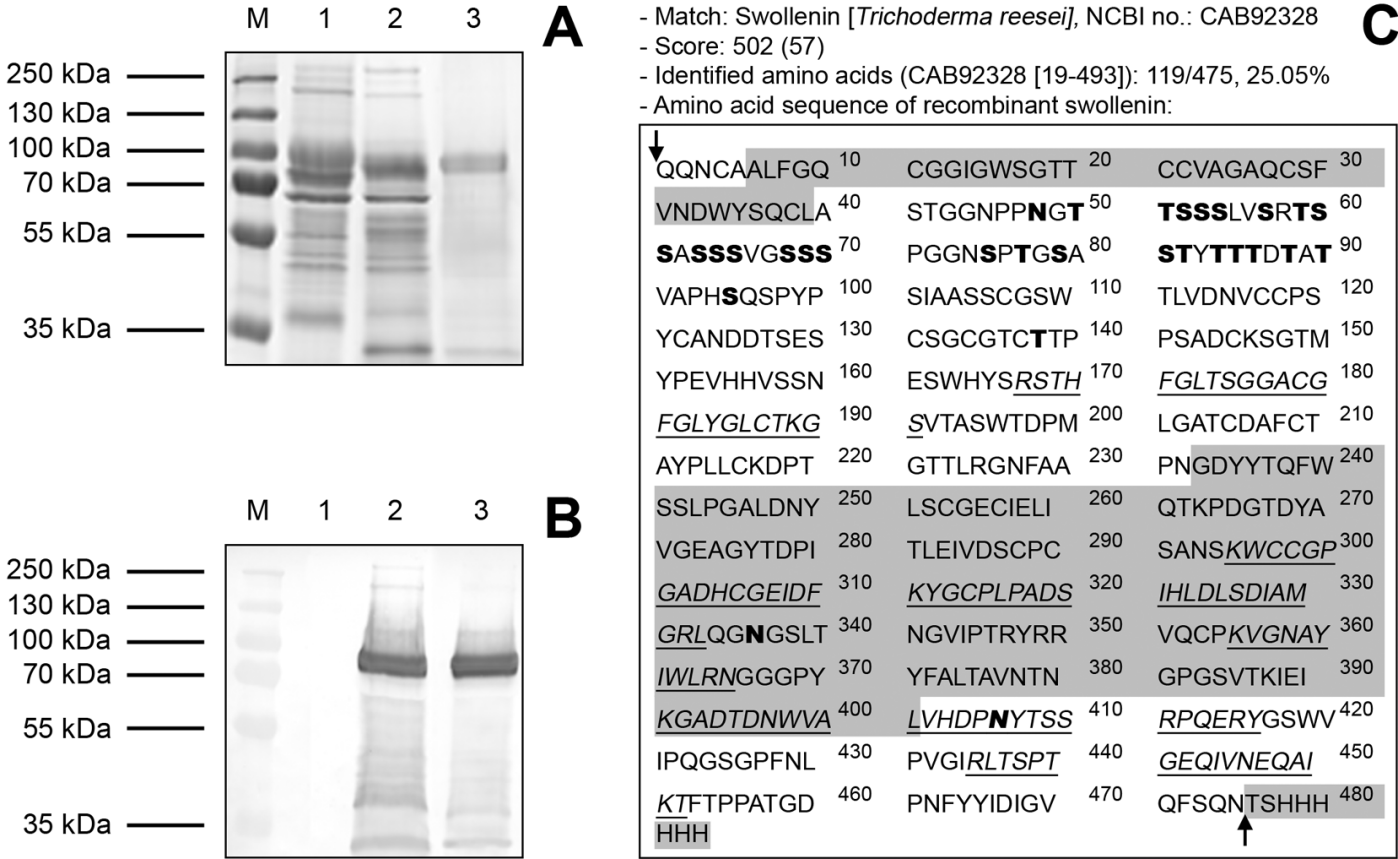
**SDS-PAGE, Western blot and mass spectrometry of swollenin produced by *Kluyveromyces lactis***. (**A**) SDS-PAGE and (**B**) Western blot: (M) Molecular mass marker, (1) filtrated culture supernatant of *K. lactis *wild type, (2) filtrated culture supernatant of *K. lactis *expressing recombinant swollenin, (3) recombinant swollenin purified by immobilized metal affinity chromatography. 12% polyacrylamide gel, the same volume of the samples (15 μL) was loaded onto the particular slots; (**C**) Mass spectrometric results and primary sequence of recombinant swollenin. The protein band (around 80 kDa) was analyzed using a mass spectrometer and the Mascot database. The detected peptides are underlined and written in italic letters. The cellulose-binding domain [6-39], expansinA domain [243-401] and His-tag [476-483] are marked in grey. Potential areas for N-glycosylation and O-glycosylation are written in bold letters. The black arrows enclose the primary sequence of the native swollenin (CAB92328) without leader peptide.

Swollenin is a cellulase-related protein and consists of an N-terminal cellulose-binding domain connected by a linker region to an expansin-homologous domain [50]. The cDNA for swollenin from *T. reesei *was used as a template to clone a recombinant His-tagged swollenin (data not shown). After cloning, the recombinant swollenin was heterologously expressed by using the yeast *K. lactis *as expression host [55]. In addition, a non-transformed *K. lactis *wild type was cultivated as a reference. As shown by SDS-PAGE (Figure 1A), the supernatants of the wild type (lane 1) and the transformed clone (lane 2) showed only a few differences in protein secretion pattern. These differences could be explained by the influence of heterologous protein expression on the native secretome of *K. lactis *[56]. However, an intense protein band at about 80 kDa could be observed in the supernatant of the transformed clone which corresponds to the size of native swollenin from *T. reesei *(about 75 kDa, 49 kDa based on the primary sequence) [50]. Furthermore, this protein band was detected as a His-tagged protein by Western blot analysis (Figure 1B). In order to quantify the putative swollenin in the supernatant of *K. lactis*, the total protein concentration was determined and a densitometric analysis of the SDS-polyacrylamide gel (Figure 1A, lane 2) was conducted. The expression level of swollenin was approximately 20 to 30 mg/L, which is comparable with the results for other recombinant proteins expressed in *K. lactis *[55,56]. With respect to recombinant swollenin, lower or comparable expression levels were achieved by using *S. cerevisiae *(25 μg/L) [50] or *A. oryzae *(50 mg/L) [53] as expression hosts. Finally, this protein was purified by immobilized metal ion affinity chromatography. According to Figure 1A and 1B, the final fraction (lane 3) showed a protein band with high purity (around 75%).

To clearly identify the protein band at about 80 kDa (Figure 1A and 1B), its amino acid sequence was determined by using mass spectrometry [57] and the Mascot search engine [58]. Figure 1C shows the results of mass spectrometry and the expected amino acid sequence of the recombinant swollenin. As shown by a high Mascot score of 502 (Figure 1C), the protein at 80 kDa was clearly identified to be a variant of swollenin from *T. reesei*. Regarding the native swollenin sequence, a protein score of greater than 57 (homology threshold) indicates identity or extensive homology (*P *< 0.05). In addition, potential N- and O-glycosylation sites were detected by using the NetNGlyc 1.0 and NetOGlyc 3.1 servers [59] (Figure 1C). Here, it should be noted that the native swollenin contains almost no N-glycosylation [50]. Therefore, the difference between the calculated molecular mass of 49 kDa, based on the primary sequence of swollenin, and the observed molecular mass of 80 kDa (Figure 1A and 1B) may be explained by O-glycosylation and other post-translational modifications. Proofs are given as follows: (i) the linker region of cellulases or cellulase-related proteins is highly O-glycosylated [60]; (ii) swollenin contains potential O-glycosylation sites within the linker region (Figure 1C); (iii) no peptides of the linker region were identified by mass spectrometry, since glycosylation alters the mass/charge ratio of the peptides (Figure 1C).

### Adsorption of swollenin

**Figure 2:**
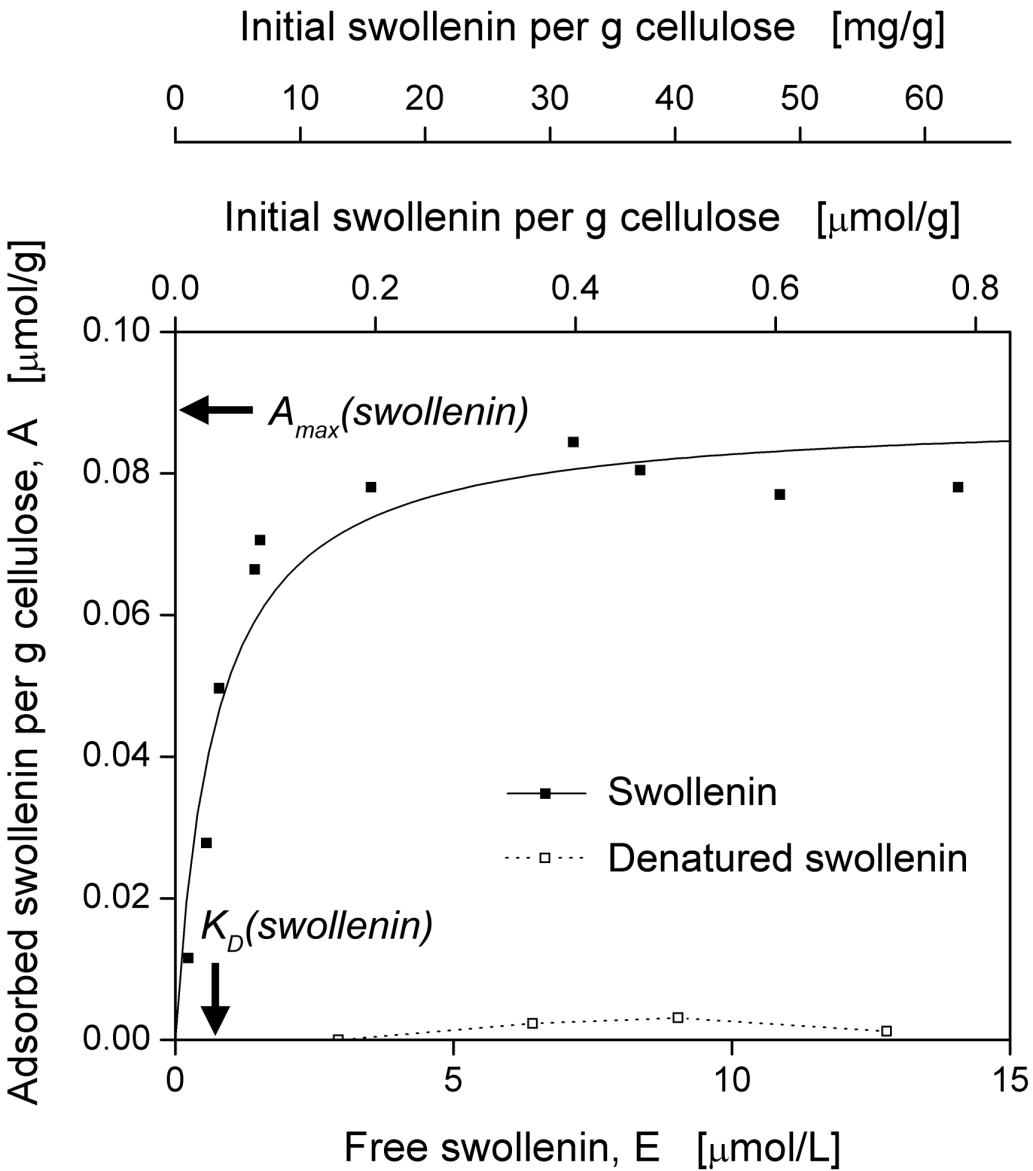
**Adsorption isotherm of purified swollenin onto filter paper**. The predicted Langmuir isotherm, according to Equation (1), is shown as a solid line (*R^2 ^*= 0.91) and corresponding parameters (including standard deviations) are: *A_max_(swollenin) *= 0.089 ± 0.006 μmol/g, *K_D_(swollenin) *= 0.707 ± 0.196 μmol/L. The initial swollenin concentration, added at the start of the incubation, is also shown for a better understanding of Figure 8; 20 g/L Whatman filter paper No.1 in 0.05 M sodium acetate buffer at pH 4.8, *T *= 45°C, *V_L _*= 1 mL, *n *= 1000 rpm, *d_0 _*= 3 mm, incubation time 2 h.

As the adsorption of proteins is a prerequisite for amorphogenesis [20], the adsorption isotherm of purified swollenin onto filter paper was determined. Preliminary adsorption kinetics showed that an incubation time of less than or equal 2 h was needed to reach equilibrium. Figure 2 illustrates that the adsorption of swollenin was a characteristic function of free swollenin concentration. After a sharp increase in adsorbed swollenin at low concentrations, a plateau was reached at higher concentrations (> 5 μmol/L). As denatured swollenin, boiled for 20 min, showed no adsorption (Figure 2), the adsorption was specific and required a functional protein structure. The Langmuir isotherm (Equation 1) provided a good fit (Figure 2, *R^2 ^*= 0.91). Corresponding parameters - the maximum swollenin adsorption per g cellulose at equilibrium, *A_max_(swollenin)*; and the dissociation constant of swollenin, *K_D_(swollenin) *- are listed in the legend of Figure 2. Similar values of *A_max _*and *K_D _*were found when analyzing the adsorption of purified cellulases onto filter paper ([61], CBH I: 0.17 μmol/g, 0.71 μmol/L; EG I: 0.17 μmol/g, 1.79 μmol/L). This may be attributed to the fact that swollenin exhibits a cellulose-binding domain with high homology to those of cellulases [62]. However, *A_max _*was lower for swollenin than for single cellulases. According to Linder *et al*. [63], single amino acid substitutions of cellulose-binding domains can lead to adsorption differences. Furthermore, catalytic domains of cellulases are known to specifically adsorb onto cellulose independently of cellulose-binding domains [41]. In addition, the difference in *A_max _*may be explained by the lower molecular mass of cellulases [64] and, therefore, a better access to internal binding sites as described for other proteins and materials [65,66].

### Pretreatment of filter paper with swollenin

**Figure 3:**
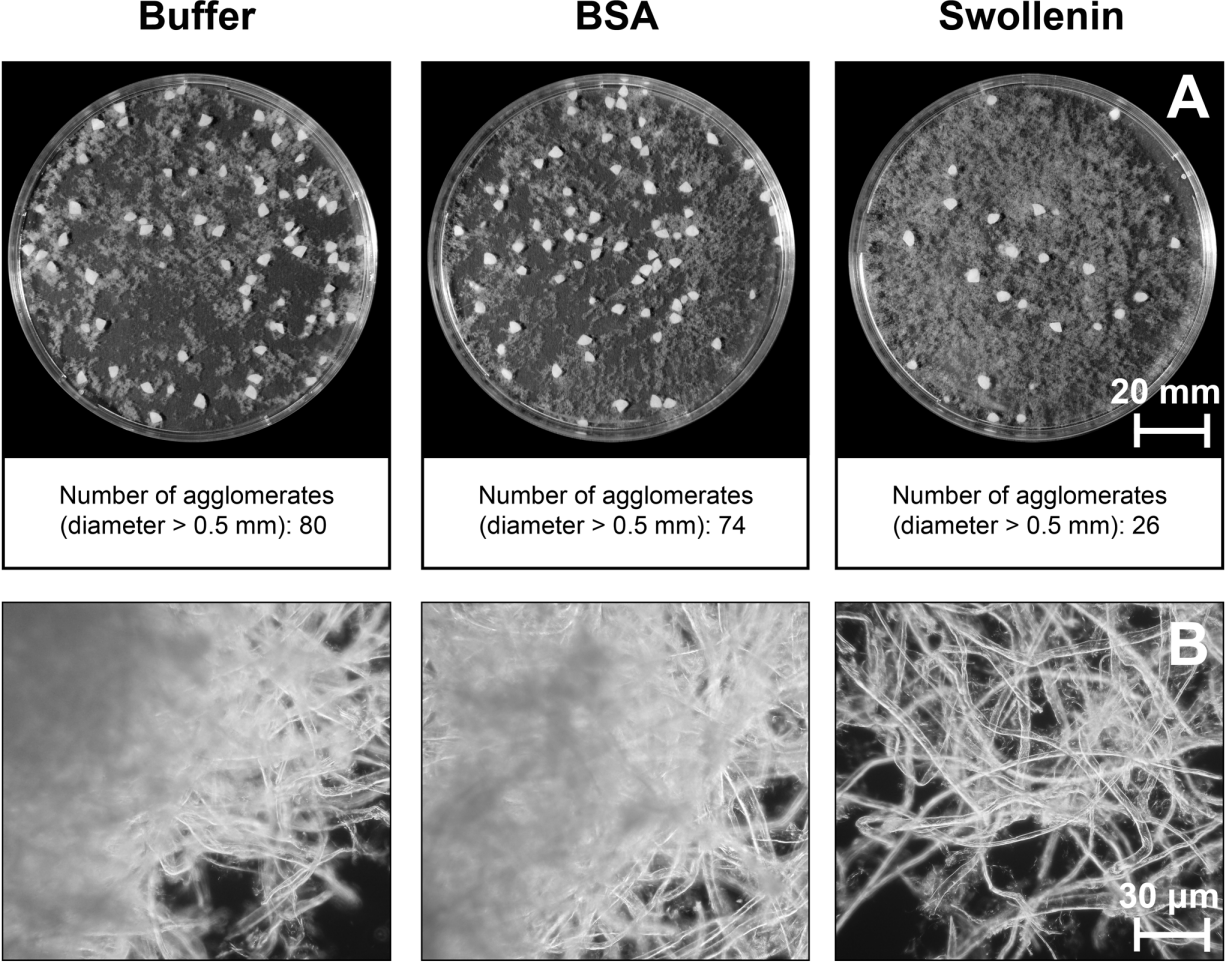
**Photography and light microscopy of filter paper after pretreatment with swollenin**. (**A**) Macroscopic pictures of pretreated filter paper in petri dishes and number of agglomerates. All pretreatments were initiated with the same initial number (80) of filter paper agglomerates (initial diameter approx. 3 mm). Number of agglomerates (> 0.5 mm) was measured by image analysis; (**B**) Light microscopy of pretreated filter paper. Eclipse E600 (Nikon); Pretreatment: 20 g/L cellulose in 0.05 M sodium acetate buffer at pH 4.8, 0.4 g/L BSA (approx. 6 μmol/L) or swollenin (approx. 5 μmol/L), *T *= 45°C, *V_L _*= 1 mL, *n *= 1000 rpm, *d_0 _*= 3 mm, incubation time 48 h.

To verify a potential effect of recombinant swollenin on cellulose, filter paper was pretreated with buffer, BSA or recombinant swollenin. Here, swollenin in an initial concentration of 20 mg per g cellulose was applied (> 80% saturation, Figure 2). It should be noted that all pretreatments were initiated with the same initial number (80) of filter paper agglomerates (initial diameter approximately 3 mm). As shown in Figure 3A, swollenin caused a deagglomeration of filter paper agglomerates (consisting of cellulose fibers). Since the cellulose fibers of a single agglomerate were separated by pretreatment with swollenin (Figure 3B), the number of bigger agglomerates obviously decreased (Figure 3A). This decrease in the number of bigger agglomerates (> 0.5 mm) was also quantified using image analysis (Figure 3A). During pretreatment, a shaken system with relatively low shear forces was applied. However, to exclude a sole mechanical effect on cellulose agglomerates due to shaking and to verify a specific effect of swollenin, filter paper was accordingly pretreated with buffer or the protein BSA (references). By contrast, the pretreatments with buffer or BSA showed much less deagglomeration (Figure 3A and 3B). Consequently, the deagglomeration was specifically caused by swollenin. As no reducing sugars were detected when using the sensitive p-hydroxy benzoic acid hydrazide assay after an incubation with swollenin for 48 h, the reduction in the number of large agglomerates was attributed to the aforementioned adsorption of swollenin onto filter paper (Figure 2) and the so-called non-hydrolytic deagglomeration [20].

**Figure 4:**
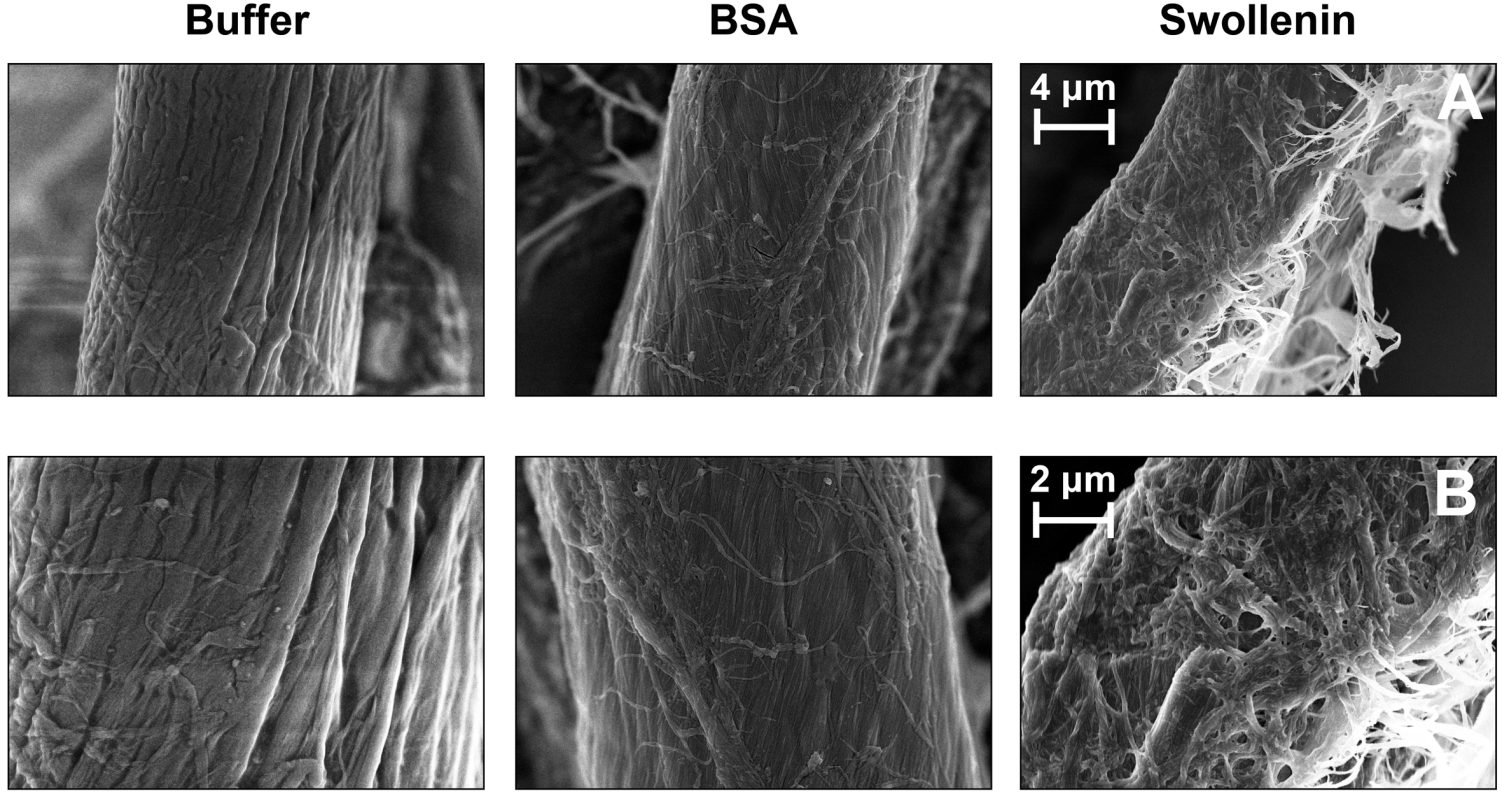
**Scanning electron microscopy of filter paper after pretreatment with swollenin**. Pictures were taken at two different magnifications (**A, B**): see scale markers; Pretreatment: 20 g/L cellulose in 0.05 M sodium acetate buffer at pH 4.8, 0.4 g/L BSA (approx. 6 μmol/L) or swollenin (approx. 5 μmol/L), *T *= 45°C, *V_L _*= 1 mL, *n *= 1000 rpm, *d_0 _*= 3 mm, incubation time 48 h. Hitachi S-5500 (Hitachi).

As described by Saloheimo *et al*. [50], swollenin is also able to disrupt and swell cotton fibers. This phenomenon results from the dispersion of cellulose microfibrils and is called amorphogenesis [20,42]. In this current study, however, the swelling of cellulose fibers was not detected when Whatman filter paper No.1 - a different substrate - was used (Figure 3B). Reasons for this may be the different structure of filter paper than that of cotton used by Saloheimo *et al*. [50] or the low resolution of light microscopy. Therefore, scanning electron microscopy was applied to visualize the effect of swollenin on cellulose microfibrils (Figure 4A and 4B). After pretreatments with buffer or BSA, the microfibrils were not dispersed, thereby resulting in a smooth and uniform surface of the whole fiber. By contrast, swollenin caused the microfibrils to disperse, thereby creating a rough and amorphic surface on the cellulose fibers. Other authors found similar results via scanning electron microscopy after treating cellulose with cellulose-binding domains of cellulases [17,45,67]. However, the results of this current study indicate that recombinant swollenin from *K. lactis *may induce amorphogenesis of cellulosic substrates.

The non-hydrolytic deagglomeration or amorphogenesis of cellulose was also described for single cellulose-binding domains of cellulases [17,45,67] and for other expansin-related proteins from *B. subtilis *[51], *A. fumigatus *[46] or *B. adusta *[52]. However, there is no detailed and quantitative analysis of different cellulosic substrates after pretreatment with non-hydrolyzing proteins, especially with regard to swollenin.

### Effect of swollenin pretreatment on the physical properties of cellulosic substrates

To analyze in detail the effect of recombinant swollenin on cellulose, different cellulosic substrates were pretreated with buffer, BSA or recombinant swollenin. After pretreatment and removal of bound proteins, the physical properties of the pretreated cellulosic substrates were analyzed by laser diffraction, cellulase adsorption studies and crystallinity measurements.

**Figure 5:**
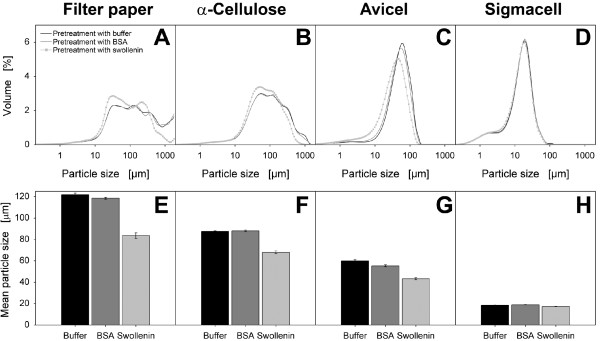
**Particle size of cellulosic substrates after pretreatment with swollenin**. (**A, B, C, D**) Volumetric particle-size distribution of pretreated cellulosic substrates: (**A**) Whatman filter paper No.1; (**B**) α-Cellulose; (**C**) Avicel PH101; (**D**) Sigmacell 101; (**E, F, G, H**) Geometric mean particle size of pretreated cellulosic substrates: (**E**) Whatman filter paper No.1; (**F**) α-Cellulose; (**G**) Avicel PH101; (**H**) Sigmacell 101. Errors are given as standard deviations; Pretreatment: 20 g/L cellulose in 0.05 M sodium acetate buffer at pH 4.8, 0.4 g/L BSA (approx. 6 μmol/L) or 0.4 g/L swollenin (approx. 5 μmol/L), *T *= 45°C, *V_L _*= 1 mL, *n *= 1000 rpm, *d_0 _*= 3 mm, incubation time 48 h. Particles (< 2 mm) were analyzed using the particle size analyzer LS13320 (Beckman Coulter).

As seen in Figure 5A-D, the cellulosic substrates showed broad and inhomogeneous particle-size distributions. Upon considering the same cellulosic substrate, the pretreatments with buffer or BSA led to no differences in particle-size distributions and in the resulting geometric mean particle sizes (Figure 5E-H). After swollenin pretreatment, however, the particle-size distributions shifted to lower values, and large cellulose agglomerates were predominantly deagglomerated to smaller particles. The bigger the initial particle size of the corresponding cellulosic substrate was, the greater the reduction in mean particle size by swollenin pretreatment was (filter paper > α-cellulose > Avicel). In the case of Sigmacell, all particle-size distributions were identical (Figure 5D), and the mean particle sizes did not change significantly due to pretreatment with swollenin (Figure 5H). This may be explained by the small initial particle size of Sigmacell and the absence of cellulose agglomerates.

Since cellulosic particle sizes (external surface areas) influence cellulose accessibility [15,19], they also affect the adsorption of cellulases [21,25] and they are an indication for the maximum cellulase adsorption [21]. To investigate if swollenin pretreatment actually affected cellulose accessibility, cellulase adsorption was analyzed after pretreatment with buffer or swollenin. According to various authors, the adsorption of total cellulase mixtures is not interpretable by simple Langmuir isotherms due to multicomponent cellulase adsorption [10,68]. Consequently, only the maximum cellulase adsorption per g cellulose *A_max_(cellulase) *was determined by applying different incubation times and a total cellulase mixture at high concentrations. Since no further increase in cellulase adsorption was detected after 1.5 h (data not shown), adsorption equilibrium was verified. According to the literature, cellulase adsorption is rapid and adsorption equilibrium is usually reached within 0.5 to 1.5 h [41,64]. Saturation of all applied cellulosic substrates was reached when using the following cellulase/cellulose ratios: ≥ 100 mg/g (in the case of filter paper or α-cellulose), ≥ 150 mg/g (Avicel), ≥ 200 mg/g (Sigmacell). Table 1 summarizes the maximum cellulase adsorption per g cellulose (adsorption capacity) onto all applied cellulosic substrates after pretreatment with buffer or swollenin. In general, the determined *A_max_(cellulase) *values are consistent with the adsorption data reported in the literature [10,41,69]. However, the pretreatment with swollenin caused a significant increase in maximum cellulase adsorption except for Sigmacell. The relative increase in cellulase adsorption between the pretreatment with swollenin and the pretreatment with buffer (filter paper > α-cellulose > Avicel > Sigmacell) showed a similar series as the relative reduction in mean particle size (filter paper > Avicel > α-cellulose > Sigmacell; Figure 5). Consequently, the increase in adsorption capacities of the swollenin-pretreated samples resulted primarily from the reduction in particle size and the corresponding increase in cellulose accessibility. However, in the case of α-cellulose, the increase in maximum cellulase adsorption was disproportionately higher. This can be explained by the effect of swollenin on other physical properties of cellulose, such as crystallinity, which may influence cellulase adsorption according to various authors [26,27]. Moreover, since all applied cellulosic substrates do not contain lignin, its influence on cellulose accessibility [31,70-72] could be neglected.

**Table 1:** Maximum cellulase adsorption onto cellulosic substrates after pretreatment with swollenin.

Substrate	Pretreatment with buffer	Pretreatment with swollenin
	*A_max_(cellulase) *(mg/g)	*A_max_(cellulase) *(mg/g)
Whatman filter paper No.1	16	31
α-Cellulose	21	35
Avicel PH101	52	73
Sigmacell 101	119	122

The coefficients of variation were below 7.5% for each value. *A_max_(cellulase) *denotes the maximum cellulase adsorption per g cellulose at equilibrium.

**Figure 6:**
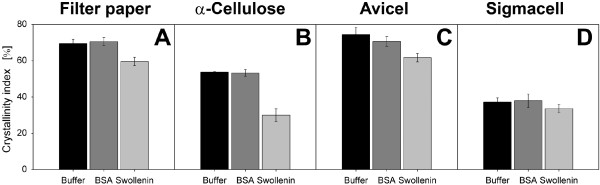
**Crystallinity index of cellulosic substrates after pretreatment with swollenin**. **(A) **Whatman filter paper No.1; (**B**) α-Cellulose; (**C**) Avicel PH101; (**D**) Sigmacell 101. Errors are given as standard deviations; Pretreatment: 20 g/L cellulose in 0.05 M sodium acetate buffer at pH 4.8, 0.4 g/L BSA (approx. 6 μmol/L) or 0.4 g/L swollenin (approx. 5 μmol/L), *T *= 45°C, *V_L _*= 1 mL, *n *= 1000 rpm, *d_0 _*= 3 mm, incubation time 48 h. Powder XRD (STOE & Cie GmbH).

To additionally determine the influence of swollenin on the crystallinity of cellulose, the crystallinity index (*CrI*) of all pretreated cellulosic substrates was analyzed by X-ray diffraction (XRD) measurements (Figure 6A-D). A recrystallization of cellulose by incubation with aqueous solutions [73,74] was not observed, because the initial *CrI *of untreated substrates was higher than that of cellulosic substrates treated with buffer (data not shown). As illustrated by Figure 6, the pretreatment with buffer or BSA caused no differences in *CrI*; the *CrI *values were identical upon considering the same cellulosic substrate. By contrast, swollenin pretreatment specifically reduced the *CrI *as follows: filter paper (-10%), α-cellulose (-22%) and Avicel (-13%). However, in the case of Sigmacell, no effect of swollenin pretreatment on *CrI *was detected (Figure 6D) which can be explained by the low initial *CrI *and the amorphous structure of Sigmacell [75]. The strongest reduction in *CrI *was recorded in the case of α-cellulose (Figure 6B). Since α-cellulose is fibrous [64] and can consist of up to 22% xylan [76], it may be more sensitive to non-hydrolytic decrystallization [77]. However, the strong reduction in the *CrI *of α-cellulose explains the disproportionate increase in maximum cellulase adsorption onto α-cellulose (Table 1), since cellulase adsorption can increase with decreasing *CrI *[26]. As reported in the literature, similar reductions in crystallinity were found by using other non-hydrolyzing proteins: (i) the *CrI *of Avicel decreased by 9% to 12% after pretreatment with single cellulose-binding domains [17]; (ii) the *CrI *of filter paper decreased by 11.8% after pretreatment with Zea h, a protein from postharvest corn stover [48]. Up to now, however, the influence of swollenin on the *CrI *of different cellulosic substrates has not been quantified. Therefore, this study provides the first proof that swollenin does induce deagglomeration of cellulose agglomerates as well as amorphogenesis (decrystallization) [20,42].

### Hydrolysis of cellulosic substrates pretreated with swollenin

**Figure 7:**
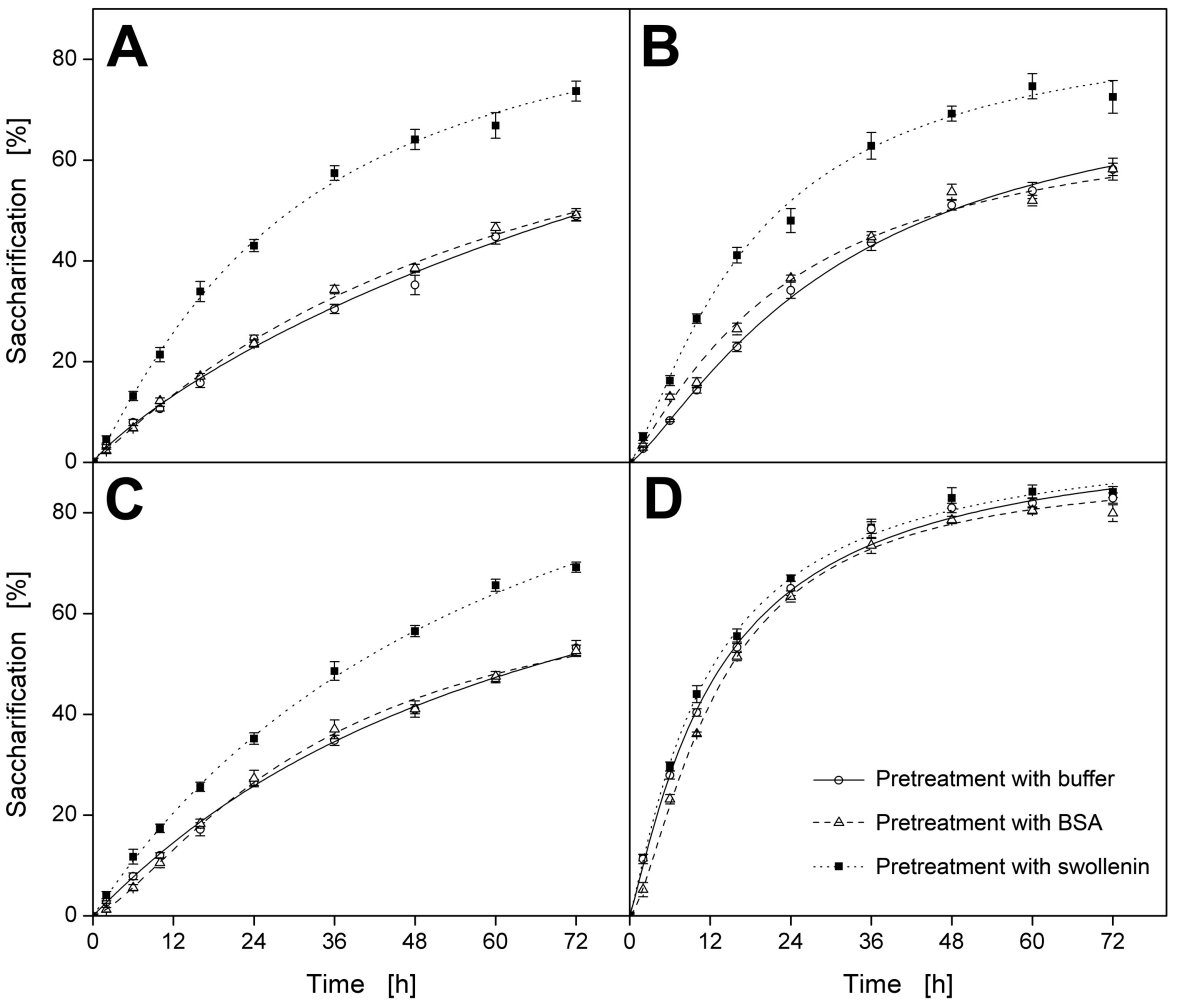
**Hydrolysis of cellulosic substrates after pretreatment with swollenin**. (**A**) Whatman filter paper No.1; (**B**) α-Cellulose; (**C**) Avicel PH101; (**D**) Sigmacell 101. Errors are given as standard deviations; Pretreatment: 20 g/L cellulose in 0.05 M sodium acetate buffer at pH 4.8, 0.4 g/L BSA (approx. 6 μmol/L) or 0.4 g/L swollenin (approx. 5 μmol/L), *T *= 45°C, *V*_*L *_= 1 mL, *n *= 1000 rpm, *d_0 _*= 3 mm, incubation time 48 h; Hydrolysis: 10 g/L pretreated cellulose in 0.05 M sodium acetate buffer at pH 4.8, 1 g/L rebuffered Celluclast^®^, *T *= 45°C, *V_L _*= 1 mL, *n *= 1000 rpm, *d_0 _*= 3 mm.

Upon using the same cellulase mixture, enzymatic hydrolysis rates are especially affected by the physical properties of the applied cellulose [10,14]. Since swollenin pretreatment affected cellulose particle size and maximum cellulase adsorption as well as crystallinity, the resulting effects on subsequent hydrolysis of all pretreated cellulosic substrates were analyzed by using rebuffered Celluclast^®^. As shown in Figure 7A-C, swollenin pretreatment significantly accelerated cellulose hydrolysis, and the saccharification after 72 h was increased. In contrast, the corresponding hydrolysis curves for buffer and BSA were almost the same by comparing the same cellulosic substrate. This is attributed to the fact that pretreatment with buffer and BSA had no significant effect on particle size (Figure 5), maximum cellulase adsorption (Table 1) or on *CrI *(Figure 6). In the case of filter paper (Figure 7A), the hydrolysis-accelerating effect of swollenin pretreatment was stronger than that for α-cellulose (Figure 7B) and Avicel (Figure 7C). This may be explained by the substantial decrease in mean particle size (Figure 5) and the strong increase in maximum cellulase adsorption (Table 1) for filter paper by swollenin pretreatment. Figure 7D shows that the hydrolysis curves of Sigmacell were almost the same, since swollenin pretreatment did not change the physical properties of Sigmacell.

**Figure 8:**
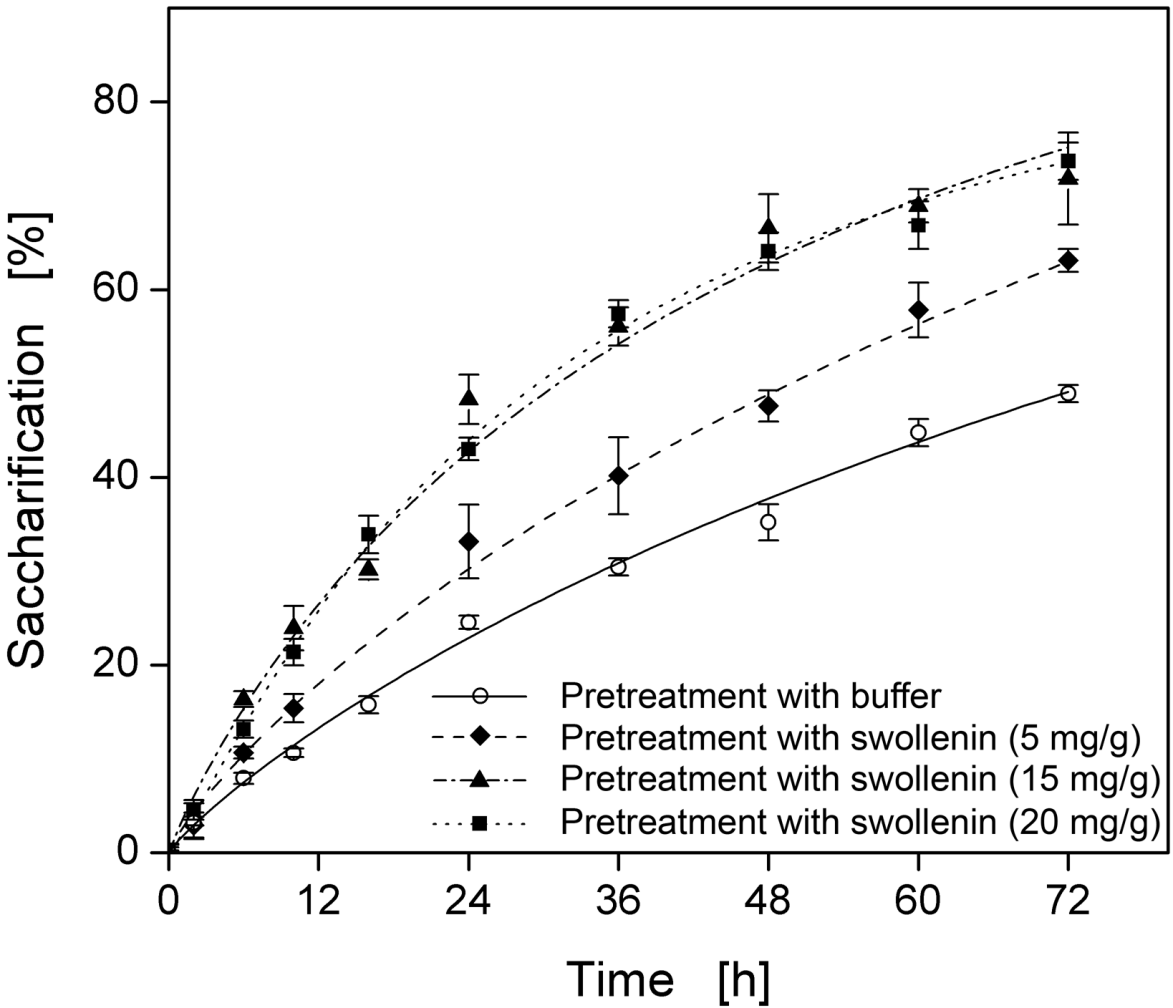
**Hydrolysis of filter paper after pretreatment with different swollenin concentrations**. Errors are given as standard deviations; Pretreatment: 20 g/L Whatman filter paper No.1 in 0.05 M sodium acetate buffer at pH 4.8, different concentrations of swollenin, *T *= 45°C, *V*_*L *_= 1 mL, *n *= 1000 rpm, *d_0 _*= 3 mm, incubation time 48 h; Hydrolysis: 10 g/L pretreated cellulose in 0.05 M sodium acetate buffer at pH 4.8, 1 g/L rebuffered Celluclast^®^, *T *= 45°C, *V_L _*= 1 mL, *n *= 1000 rpm, *d_0 _*= 3 mm.

Furthermore, the relationship between the hydrolysis-accelerating effect and the amount of swollenin applied during pretreatment was investigated (Figure 8). Compared to the aforementioned experiments (Figure 7 and 8; 20 mg swollenin per g cellulose), less swollenin (5 mg per g cellulose) caused a less accelerated hydrolysis and the final concentration of reducing sugars was 0.85-fold smaller. However, when the amount of swollenin was decreased merely from 20 mg/g to 15 mg/g, the same reducing sugar concentration was detected after 72 h. Since maximum swollenin adsorption was reached at higher initial swollenin concentrations (> 60 mg/g for 95% saturation, Figure 2), these results show that even non-saturating swollenin concentrations of 15 to 20 mg/g are sufficient for a maximum hydrolysis-accelerating effect. This may be explained as follows: (i) not all accessible cellulose-binding sites must be occupied for a maximum hydrolysis-accelerating effect; (ii) swollenin reversibly binds to cellulose, thereby performing further deagglomeration and amorphogenesis at multiple cellulose-binding sites. The reversible adsorption onto cellulose-binding sites was already reported for cellulases containing cellulose-binding domains [78].

**Figure 9:**
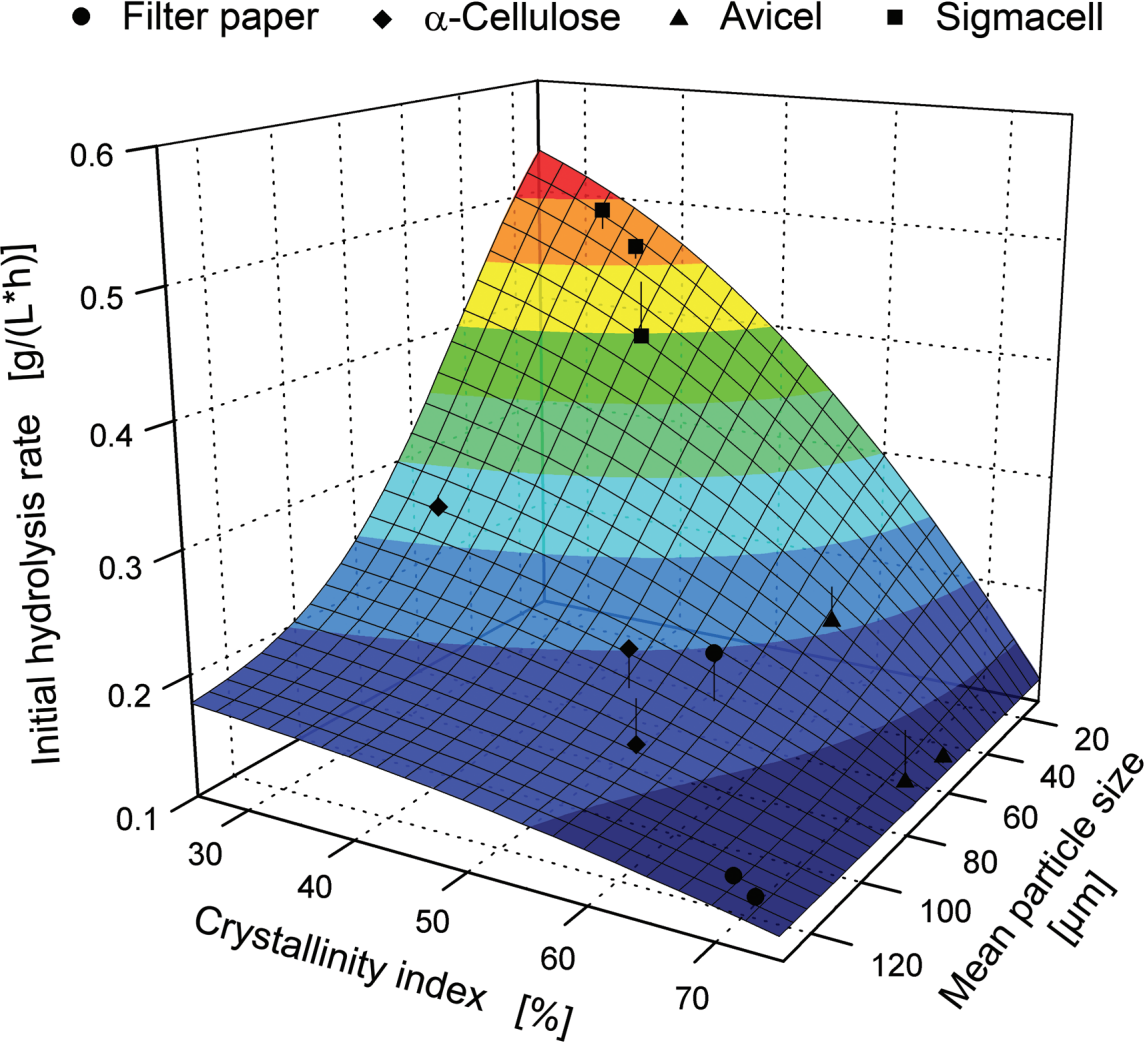
**Influence of crystallinity and mean particle size on hydrolysis of cellulosic substrates**. Data points for cellulosic substrates were obtained from Figure 5 (mean particle size), Figure 6 (crystallinity index) and Figure 7 (initial hydrolysis rate from 0 to 6 h). TableCurve 3D was used to determine an empirical surface fit (*R^2 ^*= 0.93) based on a non-linear Gaussian cumulative function.

Finally, an empirical correlation for initial hydrolysis rates based on *CrI *and mean particle size was determined for the pretreated cellulosic substrates (Figure 9). In this investigation, the correlation showed that the swollenin-induced reduction in *CrI *and particle size resulted in high cellulose hydrolysis rates. Furthermore, Figure 9 illustrates the aforementioned differences in cellulose hydrolysis rates (Figure 7) for various substrates and pretreatments. In addition, it confirms the findings of other authors: (i) since smaller cellulose particle sizes lead to increased cellulase adsorption [25] (see previous section), hydrolysis rates increase with decreasing cellulose particle size [22-24]; (ii) since a reduction in *CrI *leads to increased cellulase adsorption and higher reactivity of adsorbed cellulases, hydrolysis rates correlate inversely with the *CrI *of the applied cellulose [24,26]. It should be noted that Figure 9 shows an empirical correlation for the conducted hydrolysis experiments. By applying other concentrations or types of cellulases and cellulosic substrates, different physical properties of the substrate (for example, porosity, [79]) might predominate.

## Conclusions

Recombinant swollenin was easily produced with the yeast *K. lactis *and purified by affinity chromatography. Additionally, the adsorption of swollenin onto cellulose was quantified for the first time, and its adsorption parameters were comparable to those of individual cellulases. The pretreatment with swollenin caused a significant decrease in particle size as well as in crystallinity of the cellulosic substrates, thereby substantially increasing maximum cellulase adsorption. Moreover, pretreatment of the cellulosic substrates with swollenin - even in non-saturating concentrations - significantly accelerated the hydrolysis. By correlating particle size and crystallinity with initial hydrolysis rates, it could be shown that high initial hydrolysis rates resulted from the swollenin-induced reduction in particle size and crystallinity. Consequently, this study shows an efficient means to produce recombinant swollenin with the robust yeast *K. lactis*. Moreover, this study shows that swollenin induces deagglomeration of cellulose agglomerates as well as amorphogenesis (decrystallization). For the first time, this study quantifies and elucidates in detail how swollenin affects cellulosic substrates and their hydrolysis.

A pretreatment of cellulosic substrates has been presented here which is simply based on the incubation of recombinant swollenin under mild conditions. Since the enzymatic hydrolysis of cellulose is a rate-limiting processing step in biorefineries [41], this pretreatment could significantly improve hydrolysis rates. To exclude possible side effects between swollenin and cellulase, swollenin pretreatment was performed as a separate step within this study. In future studies, swollenin should be directly added during cellulose hydrolysis. Since standard assays are missing for deagglomeration, amorphogenesis and for the comparison of different non-hydrolyzing proteins, this study may serve as an initial means to establish such assays.

## Methods

### Cellulosic substrates and cellulases

The cellulosic substrates Whatman filter paper No.1, α-cellulose, Avicel PH101 and Sigmacell 101 were purchased from Sigma-Aldrich (MO, USA). Physical properties and product information have been summarized by various authors [10,64]. Agglomerates of Whatman filter paper No.1 were prepared by using a hole-punch and quartering the resulting filter paper discs. The final filter paper agglomerates had an average diameter of approximately 3 mm. The cellulase preparation Celluclast^® ^1.5 L (Novozymes, Bagsværd, DK) - a filtrated culture supernatant of *T. reesei *[80] - was used for the hydrolysis of the pretreated cellulosic substrates. According to various authors, Celluclast^® ^contains CBHs (Cel7A and Cel6A), EGs (for example, Cel7B and Cel5A) as well as β-glucosidases [81,82]. To remove salts, sugars and other interfering components, Celluclast^® ^was previously rebuffered with an Äkta FPLC (GE Healthcare, Little Chalfont, UK). Celluclast^® ^was loaded on Sephadex G-25 Fine (2.6 cm × 10 cm, GE Healthcare), and 0.05 M sodium acetate (pH 4.8) was used as a running buffer at 110 cm/h. Sephadex G-25 Fine exhibits an exclusion limit of 1-5 kDa which is comparable to the molecular mass cut-off of dialysis membranes for protein desalting. Since cellulases have a molecular mass of > 25 kDa [62,83,84], the mixture of cellulases was not changed during rebuffering. Chromatography was conducted at room temperature, and the automatically collected fractions were directly cooled at 4°C. To determine specific filter paper activities, different dilutions of Celluclast^® ^and the rebuffered Celluclast^® ^- applied for all hydrolysis experiments - were tested according to Ghose [85]. Here, the following specific filter paper activities (per g protein) were measured: 201 U/g (Celluclast^®^) and 279 U/g (rebuffered Celluclast^®^).

### Genetic engineering for recombinant swollenin

The below-mentioned cloning procedure was designed for secreted protein expression according to the *K. lactis *Protein Expression Kit (New England Biolabs, MA, USA). The cDNA of the swollenin-coding region was synthesized by reverse-transcription PCR using mRNA isolated from *T. reesei *QM9414 (swo1 gene [GenBank: AJ245918], protein sequence [GenBank: CAB92328]) and reverse transcriptase (M-MLV, Promega, WI, USA) according to the manufacturer's protocol. Specific primers were applied to synthesize a cDNA starting from the 19^th ^codon of the swollenin-coding region and, therefore, missing the secretion signal sequence of *T. reesei *[50]. By using the aforementioned primers, SalI and SpeI restriction sites were added upstream and downstream of the swollenin-coding region, respectively. The amplified cDNA was cloned into the pCR2.1-TOPO vector (Invitrogen, CA, USA) according to the manufacturer's protocol. After DNA sequencing and isolation of a correct clone, the DNA was excised from pCR2.1-TOPO and cloned into the pKLAC1-H vector using XhoI and SpeI restriction enzymes (New England Biolabs, MA, USA) according to the manufacturer's protocol. The pKLAC1-H is a modified version of the integrative pKLAC1 vector (New England Biolabs; [GenBank: AY968582]). The pKLAC1 - developed by Colussi and Taron [55] - exhibits the α-mating factor signal sequence and can be used for the expression and secretion of recombinant proteins in *K. lactis *[55]. The pKLAC1-H was constructed by including an additional SpeI restriction site directly followed by a His-tag coding sequence (6xHis) between the XhoI and AvrII restriction sites of pKLAC1. The DNA sequence of the final pKLAC1-H construct (containing the DNA coding for recombinant swollenin) is shown in Additional file 1. Moreover, the final amino acid sequence of recombinant swollenin (without the α-mating factor signal sequence) is given in Figure 1C.

### Expression and purification of recombinant swollenin

All below-mentioned transformation, selection and precultivation procedures - developed by Colussi and Taron [55] - were performed according to the manufacturer's protocol (*K. lactis *Protein Expression Kit, New England Biolabs). After cloning, *K. lactis *GG799 cells were transformed with pKLAC1-H (containing the DNA coding for recombinant swollenin), and transformed clones were selected (acetamide selection). One clone was precultivated in YPGal (yeast extract, peptone and galactose) medium, consisting of 20 g/L galactose, 20 g/L peptone and 10 g/L yeast extract - all media components were purchased from Carl Roth (Karlsruhe, Germany). After inoculation with 2.5 mL of the preculture, the main culture was cultivated in triplicates in 2 L Erlenmeyer flasks with YPGal medium under the following constant conditions: temperature *T *= 30°C, total filling volume *V_L _*= 250 mL, shaking diameter *d_0 _= *50 mm, shaking frequency *n *= 200 rpm. Additionally, a non-transformed *K. lactis *wild type was cultivated as a reference. After incubation for 72 h, the main cultures were centrifuged (6000 g, 20 min, 4°C), and the pooled supernatants of the triplicates were treated with endoglycosidase H_f _by using 20 U per μg protein for 12 h [50] according to the manufacturer's protocol without denaturation (New England Biolabs). Afterwards, the protein solution was concentrated 100-fold at 4°C using a Vivacell 100 ultrafiltration system with a molecular mass cut-off of 10 kDa (Sartorius Stedim Biotech, Göttingen, Germany). For affinity chromatography, the recombinant swollenin was previously rebuffered using Sephadex G-25 Fine (2.6 cm × 10 cm, GE Healthcare) at 110 cm/h with a running buffer (pH 7.4) consisting of 0.05 M sodium dihydrogen phosphate, 0.3 M sodium chloride and 0.01 M imidazole. The rebuffered sample was loaded on Ni Sepharose 6 Fast Flow (1.6 cm × 10 cm; GE Healthcare) at 120 cm/h. The bound swollenin was eluted with the aforementioned running buffer, containing 0.25 M imidazole.

### SDS-PAGE and Western blot analysis

SDS-PAGE and Western blot analysis were applied to analyze the purity and to identify the recombinant swollenin. Novex 12% polyacrylamide Tris-Glycine gels (Invitrogen), and samples were prepared according to the manufacturer's protocol. The Plus Prestained Protein Ladder (Fermentas, Burlington, CA, USA) was used as a molecular mass marker. Finally, the proteins were stained with Coomassie Brilliant Blue and analyzed densitometrically [86] using the scanner Perfection V700 (Epson, Suwa, Japan). The molecular mass and purity of swollenin was determined using the software TotalLab TL100 (Nonlinear Dynamics, Newcastle, UK). For Western blot analysis, gels were blotted onto a nitrocellulose membrane (Whatman, Springfield Mill, UK) according to the manufacturer's protocol (Invitrogen). The membranes were blocked at room temperature with 50 g/L skim milk dissolved in phosphate buffered saline containing 0.5 g/L Tween-20 (PBST) for 30 min. To detect the recombinant swollenin, the membranes were incubated at room temperature for 1.5 h with a rabbit polyclonal antibody against His-tag (Dianova, Hamburg, Germany) diluted 1:10,000 in PBST. After the membrane was washed thrice with PBST, it was incubated with alkaline phosphatase conjugated goat anti-rabbit IgG (Dianova) diluted 1:5,000 in PBST at room temperature for 1 h. Finally, bound antibodies were visualized by incubating the membrane for 5 min with nitro blue tetrazolium/5-Bromo-4-chloro-3-indolyl phosphate (NBT/BCIP) diluted 1:100 in phosphatase buffer (100 mM Tris-HCl, 100 mM NaCl, 5 mM MgCl_2_, pH 9.6).

### Measurement of protein concentration

Protein concentrations were analyzed with the bicinchoninic acid assay [87] using the BCA Protein Assay Kit (Thermo Fisher Scientific, MA, USA) and BSA as a standard. Depending on the protein concentration of the samples, the standard procedure (working range: 0.02 to 2 g/L) or the enhanced procedure (working range: 0.005 to 0.25 g/L) was performed according to the manufacturer's protocol. The absorbance at 562 nm was measured with a Synergy 4 microtiter plate reader (BioTek Instruments, VT, USA). To quantify swollenin in the culture supernatant of *K. lactis*, the bicinchoninic acid assay was combined with the aforementioned SDS-PAGE (including densitometric analysis). Here, total protein concentrations were determined and multiplied with the ratio of swollenin to total protein (purity).

### Mass spectrometry and glycosylation analysis

Mass spectrometry was applied to identify the expressed and purified recombinant swollenin. The protein band (approximately 80 kDa) was excised from the SDS-polyacrylamide gel, washed in water, reduced with dithiothreitol, alkylated with iodoacetamide, and digested with trypsin [88]. Peptide analysis was carried out using a nanoHPLC (Dionex, Germering, Germany) coupled to an ESI-QUAD-TOF-2 mass spectrometer (Waters Micromass, Eschborn, Germany) as previously described [89]. The Mascot algorithm (Matrix Science, London, UK) was used to correlate the mass spectrometry data with amino acid sequences in the Swissprot database. Thereby, the sequences of the analyzed peptides could be identified, and, ultimately, protein matches could be determined. The Mascot score is derived from the ions scores of the detected peptides matching the peptides in the database and reflects a non-probabilistic basis for ranking protein hits [90]. By using this database, the peptide mass tolerance was set at ± 0.3 Da. Additionally, the following modifications to the amino acids in brackets were allowed: carbamidomethyl (C), carboxymethyl (C), oxidation (M), propionamide (C). Moreover, potential areas for N-glycosylation and O-glycosylation were identified by using the NetNGlyc 1.0 and NetOGlyc 3.1 servers http://www.cbs.dtu.dk/services/[59].

### Adsorption experiments

Adsorption experiments were performed in 0.05 M sodium acetate buffer (pH 4.8) using 20 g/L untreated filter paper and various concentrations (0.05 to 1.25 g/L) of purified swollenin. Solutions with filter paper and solutions with swollenin were preincubated separately at 45°C for 10 min, and experiments were started by mixing both solutions. The final mixtures were incubated in 2 mL Eppendorf tubes on a thermomixer MHR23 (simultaneous shaking and temperature control; HLC Biotech, Bovenden, Germany) under the following constant conditions for 2 h: *T *= 45°C, *V_L _*= 1 mL, *d_0 _= *3 mm, *n *= 1000 rpm. The shaking frequency was chosen to ensure the complete suspension of cellulose particles [64,91]. Three different blanks were incubated similarly: (i) without swollenin, (ii) without filter paper, or (iii) without filter paper and without swollenin. The incubation was stopped by centrifugation (8000 g, 1 min), and the supernatants were immediately analyzed for unbound swollenin by using the bicinchoninic acid assay. The adsorbed swollenin concentration was calculated as the difference between initial (blanks) and unbound swollenin concentration. Adsorption isotherm parameters were determined using the Langmuir isotherm [92]:

(1) $$A=\frac{A_{\max}\cdot E}{K_{D}+E}$$

in which *A *denotes the amount of adsorbed protein per g cellulose (μmol/g), *A_max_*, the maximum protein adsorption per g cellulose at equilibrium (μmol/g), *E*, the free protein concentration (μmol/L), and *K_D_*, the dissociation constant (μmol/L). Within the literature [61], the association constant *K_A _*(L/μmol) is sometimes used instead of the dissociation constant *K_D_*.

To analyze the effect of swollenin pretreatment (see below) on cellulase adsorption [44,93], the maximum cellulase adsorption was also determined by incubating various concentrations (0.7 to 2.5 g/L) of rebuffered Celluclast^® ^with 10 g/L pretreated cellulosic substrates. Here, all incubations were conducted under the aforementioned conditions for 1 h, 1.5 h and 2 h.

### Pretreatment with swollenin

Pretreatment experiments were performed with 20 g/L cellulosic substrates and various concentrations of swollenin in 0.05 M sodium acetate buffer (pH 4.8). The mixtures were incubated as triplicates in 2 mL Eppendorf tubes on a thermomixer under the following constant conditions: *T *= 45°C, *V_L _*= 1 mL, *d_0 _= *3 mm, *n *= 1000 rpm. To exclude a sole mechanical effect on cellulosic substrates due to shaking and to verify a specific effect of swollenin, blanks without swollenin (buffer) or with 0.4 g/L BSA instead of swollenin were incubated similarly. To detect a possible hydrolytic activity of recombinant swollenin, the sensitive p-hydroxy benzoic acid hydrazide assay [94] was applied by using glucose as a standard. After incubation for 48 h, the supernatants of the pretreatment solution were analyzed and the absorbancies were measured at 410 nm in a Synergy 4 microtiter plate reader. Subsequently, all cellulosic samples were washed to remove adsorbed proteins. Therefore, the mixtures were centrifuged (14,000 × *g*, 10 min, 4°C), and the cellulosic pellets were washed four times with 800 μL 0.05 M citrate buffer (pH 10) [95], and once with 800 μL distilled water. Finally, the triplicates were pooled. According to Zhu *et al*. [95], citrate buffer (pH 10) is an appropriate washing solution, and a single washing step with 0.05 M citrate buffer (pH 10) leads to a desorption efficiency of 61% in case of fungal cellulases and Avicel. Since no acids or bases are formed during the washing procedure, the weak buffer capacity of citrate buffer at pH 10 can be neglected. In this study, the washing procedure was conducted four times to ensure a high desorption of swollenin. The measurements of protein concentration in the washing supernatants - by applying the aforementioned bicinchoninic acid assay (working range starting from 0.005 g/L) - showed that swollenin desorbed almost completely. Already after three washing steps, a total swollenin desorption efficiency of > 90% was achieved.

### Photography and microscopy

Photography and microscopy were applied to visualize the effect of swollenin pretreatment on filter paper. After pretreatment with buffer, BSA or swollenin, the different filter paper solutions were transferred into petri dishes, the particles were evenly distributed and images were taken with an Exilim EX-FH100 camera (Casio, Tokyo, Japan). Afterwards, the number of filter paper agglomerates (> 0.5 mm) was determined by image analysis using the software UTHSCSA ImageTool 3.0 (freeware) and a ruler as a reference. Light microscopic pictures were taken with an Eclipse E600 (Nikon, Tokyo, Japan). Additionally, scanning electron microscopy was performed using a Hitachi S-5500 (Hitachi, Tokyo, Japan) and a field emission of 5 kV. All washed filter paper samples were covered with a layer of carbon (3 nm) and, subsequently, with a layer of PtPd (3 nm, 80% to 20%). The images were taken by using secondary electrons.

### **Laser diffraction and X-ray diffraction**

The particle-size distributions of all pretreated cellulosic substrates were measured by laser diffraction [96] using a LS13320 (Beckman Coulter, CA, USA). In the case of filter paper, particles with an average diameter of greater than 0.75 mm were manually removed before laser diffraction to exclude a disturbance of measurement signals. The geometric mean particle size was calculated using the software LS 5.01 (Beckman Coulter). Moreover, the *CrI *was determined by powder XRD. XRD patterns were obtained using a STOE STADI P transmission diffractometer (STOE & Cie GmbH, Darmstadt, Germany) in Debye-Scherrer geometry (Cu*Kα *radiation, *λ *= 1.54060 Å) with a primary monochromator and a position-sensitive detector. Thereby, XRD patterns were collected with a diffraction angle 2*θ *from 10° to 30° (increments of 0.01°) and a counting time of 6 s per increment. The sample was adhered to a polyester foil (biaxially-oriented polyethylene terephthalate) by using a dilute solution of glue. After drying the sample in open-air, the sample was covered with a second polyester foil. This set was then fixed in a sample holder. To improve statistics and level out sample orientation effects, the sample was rotated at around 2 Hz during XRD measurement. The *CrI *was calculated using the peak height method [28] and the corresponding equation:

(2) $$CrI=\frac{I_{002}-I_{AM}}{I_{002}}$$

where *I_002 _*is the maximum intensity of the crystalline plane (002) reflection (2*θ = *22.5°) and *I_AM _*is the intensity of the scattering for the amorphous component at about 18° in cellulose-I [97]. Here, it should be noted that there are several methods for calculating *CrI *from XRD data and these methods can provide significantly different results [28,70]. Although the applied peak height method produces *CrI *values that are higher than those of other methods, it is still the most commonly used method and ranks *CrI *values in the same order as the other methods [28].

### Hydrolysis experiments and dinitrosalicylic acid assay

Hydrolysis experiments with 10 g/L pretreated cellulosic substrate and 1 g/L rebuffered Celluclast^® ^were conducted in 0.05 M sodium acetate buffer (pH 4.8). The mixtures were incubated as triplicates in 2 mL Eppendorf tubes on a thermomixer under the following constant conditions: *T *= 45°C, total filling *V_L _*= 1 mL, *d_0 _= *3 mm, *n *= 1000 rpm. In general, attention has to be paid to cellulase inactivation, which would reduce the final yield of cellulose hydrolysis [98]. In this current study, however, a shaken system with relatively low shear forces was applied. According to Engel *et al*. [99], rebuffered Celluclast^® ^is stable under the applied incubation conditions, so that cellulase inactivation could be neglected. The shaking frequency was chosen to ensure the complete suspension of cellulose particles [64,91]. Thus, mass transfer limitations are excluded, and the whole cellulose particle surface becomes accessible to the cellulases, thereby optimizing cellulase adsorption and activity [64]. Three different blanks were incubated similarly: (i) without cellulase, (ii) without substrate, or (iii) without substrate and without cellulase. The dinitrosalicylic acid assay [100] was applied to quantify the reducing sugars released during hydrolysis by using glucose as a standard. After defined time intervals, samples were taken, and the hydrolysis was stopped (10 min, 100°C). According to Wood and Bhat [101], low reducing sugar concentrations were quantified by adding 1.25 g/L glucose to the samples. The absorbancies were measured at 540 nm in a Synergy 4 microtiter plate reader. Since the dinitrosalicylic acid assay exhibits a lower sensitivity towards cellobiose than glucose, reducing sugar concentrations may be underestimated when glucose is used as a standard and β-glucosidase is not in excess [102]. However, under the applied hydrolysis conditions, cellobiose did not accumulate (the highest cellobiose to glucose ratio was measured in the case of Sigmacell after 10 h at 0.12) and, therefore, this underestimation was minimal and the addition of β-glucosidase was not needed. Initial hydrolysis rates (g/(L*h)) were calculated by applying a linear fit to the reducing sugar concentration data from 0 to 6 h.

### Computational methods

Parameters (including standard deviations) of the adsorption model were calculated by nonlinear, least squares regression analysis using MATLAB R2010 (The MathWorks, Natick, USA). TableCurve 3D 4.0 (Systat Software, San Jose, CA, USA) was used to empirically correlate *CrI *and mean particle size with initial hydrolysis rates via the non-linear Gaussian cumulative function:

(3) z=GCUMX(a,b,c)+GCUMY(d,e,f)+GCUMX(g,b,c)⋅GCUMY(1,e,f)

in which *a, b, c, d, e, f *and *g *denote the various fitting parameters of the non-linear Gaussian cumulative function (-).

## List of abbreviations

*a*: non-linear Gaussian cumulative function parameter (-); *A*: adsorbed protein per g cellulose (μmol/g); *A_max_*: maximum protein adsorption per g cellulose at equilibrium (μmol/g or mg/g); *b*: non-linear Gaussian cumulative function parameter (-); BSA: bovine serum albumin; *c*: non-linear Gaussian cumulative function parameter (-); CBH: cellobiohydrolase; *CrI*: crystallinity index (%); *d*: non-linear Gaussian cumulative function parameter (-); *d_0_*: shaking diameter (mm); *e*: non-linear Gaussian cumulative function parameter (-); *E*: free protein concentration (μmol/L); EG: endoglucanase; *f*: non-linear Gaussian cumulative function parameter (-); *g*: non-linear Gaussian cumulative function parameter (-); *I_002_*: maximum intensity of the crystalline plane (002) reflection (1/s); *I_AM_*: XRD scattering for the amorphous component at 18^o ^in cellulose-I (1/s); *K_A_*: association constant (L/μmol) *K_D_*: dissociation constant (μmol/L); *λ*: wavelength (Å); *n*: shaking frequency (rpm); NBT/BCIP: nitro blue tetrazolium/5-Bromo-4-chloro-3-indolyl phosphate; *P*: probability for significant scores (protein matching) (-); PBST: phosphate buffered saline containing Tween-20; *R^2^*: coefficient of determination (-); *T*: temperature (°C); *θ*: diffraction angle (°); *V_L_*: filling volume (mL); XRD: X-ray diffraction; YPGal: medium containing yeast extract, peptone and galactose.

## Competing interests

The authors declare that they have no competing interests.

## Authors' contributions

GJ designed and carried out experiments, analyzed results and wrote the manuscript. MG carried out the cloning of swollenin. FD carried out the pretreatment with swollenin (incl. analysis) and subsequent hydrolysis. RR carried out the measurements of *CrI*. HB carried out scanning electron microscopy. UC and AS reviewed the manuscript. RF and JB coordinated the study and reviewed the manuscript. All authors read and approved the final manuscript.

## Supplementary Material

Additional file 1 **DNA sequence of pKLAC1-H construct (containing the DNA coding for recombinant swollenin)**. Click here for file
