# Supplementary material for: How recombinant swollenin from Kluyveromyces lactis affects cellulosic substrates and accelerates their hydrolysis
Source: Biotechnol Biofuels. 2011 Sep 23;4:33. doi: 10.1186/1754-6834-4-33 (PMC3203333; doi:10.1186/1754-6834-4-33)
Supplement: Additional file 1 — DNA sequence of pKLAC1-H construct (containing the DNA coding for recombinant swollenin). [file 1754-6834-4-33-S1.pdf]

DNA sequence of pKLAC1-H construct (containing the DNA coding for recombinant swollenin)

The pKLAC1-H is a modified version of the integrative pKLAC1 vector (New England Biolabs, MA, USA; NCBI no. AY968582). The pKLAC1 vector - developed by Colussi *et al.* [55] - can be used for the expression and secretion of recombinant proteins in *K. lactis* [55]. The pKLAC1-H was constructed by including an additional SpeI restriction site directly followed by a His-tag coding sequence (6 x His) between the XhoI and AvrII restriction sites of pKLAC1.

The cloning procedure (cloning of swollenin from *T. reesei*, NCBI no. [swo1 gene]: AJ245918, NCBI no. [protein sequence]: CAB92328) was designed for secreted protein expression according to the *K. lactis* Protein Expression Kit (New England Biolabs, MA, USA). A detailed description of the cloning procedure is given in the main manuscript.

General composition:

- 1-13: pKLAC1
- 14-262: α-mating factor signal sequence
- 263-268: Kex protease cleavage site
- 269-1720: Recombinant swollenin (incl. His-tag coding sequence and stop codon)
- 1721-10516: pKLAC1

Detailed composition:

- 14-16: Start codon **(bold)**
- 257-262: XhoI / SalI hybrid cloning site - CTCGAC **(bold, underlined)**
- 1694-1699: SpeI cloning site – ACTAGT **(bold, underlined)**
- 1700-1717: His-tag coding sequence, 6 x His **(bold, italic)**
- 1718-1720: Stop codon **(bold)**
- 1721-1726: AvrII cloning site – CCTAGG **(bold, underlined)**

1 AAGCTTGAAAAAA**ATG**AAATTCTCTACTATATTAGCCGCATCTACTGCTT
TAATTTCCGTTGTTATGGCTGCTCCAGTTTCTACCGAAACTGACATCGAC
GATCTTCCAATATCGGTTCCAGAAGAAGCCTTGATTGGATTCATTGACTT
AACCGGGGATGAAGTTTCCTTGTTGCCTGTTAATAACGGAACCCACACTG
GTATTCTATTCTTAAACACCACCATCGCTGAAGCTGCTTTCGCTGACAAG
251 GATGAT**CTCGAC**AAAAGACAGCAGAATTGCGCAGCATTATTTGGCCAATG
TGGAGGCATAGGGTGGTCCGGCACCACATGTTGCGTTGCTGGCGCCCAGT
GCAGTTTTGTCAATGACTGGTACTCCCAGTGCCTTGCGTCAACCGGCGGA
AACCCCCCAAACGGAACAACTTCCTCTAGCTTGGTTTCACGGACGTCGTC
AGCATCCTCATCCGTCGGCTCGTCTTCACCCGGCGGCAACTCACCAACTG
501 GCAGTGCTTCCACCTACACAACCACAGATACAGCTACCGTGGCTCCTCAT
TCGCAGTCTCCTTACCCCAGCATTGCCGCATCCAGTTGCGGATCGTGGAC
CCTCGTGGATAATGTTTGCTGCCCATCATATTGTGCTAATGATGACACAT
CCGAGTCATGCTCAGGCTGCGGTACCTGCACTACGCCGCCCTCGGCGGAC
TGCAAATCCGGAACCATGTATCCAGAGGTCCATCACGTATCCAGCAACGA
751 GAGCTGGCACTACAGTAGATCAACCCACTTTGGCCTAACGAGCGGCGGGG
CCTGTGGCTTTGGCCTGTACGGTCTCTGCACAAAGGGCAGTGTTACAGCC
AGCTGGACGGATCCCATGCTTGGCGCGACGTGTGACGCTTTTTGTACAGC
GTATCCCCTGCTTTGCAAAGACCCTACCGGCACTACCCTTCGTGGCAACT
TCGCAGCTCCAAACGGCGATTACTACACCCAATTCTGGTCCTCGTTGCCA
1001 GGAGCCCTCGATAACTACCTGTCCTGCGGCGAGTGCATTGAGCTGATACA
AACAAAGCCCGATGGGACCGATTATGCTGTCGGAGAAGCCGGCTACACGG
ATCCAATTACTCTCGAGATTGTGGACAGCTGCCCGTGCAGCGCGAACTCC
AAGTGGTGCTGTGGTCCGGGCGCCGATCATTGCGGAGAGATCGACTTCAA
ATACGGCTGTCCTCTTCCTGCTGACAGCATTCATCTCGACCTGTCAGACA
1251 TTGCCATGGGCCGTTTGCAGGGCAATGGATCACTAACCAATGGCGTCATC
CCGACTCGATATAGAAGAGTCCAATGCCCCAAAGTTGGGAACGCCTACAT
TTGGCTTCGAAATGGCGGAGGGCCTTACTATTTTGCTCTCACGGCAGTCA
ACACCAACGGACCGGGCTCAGTCACCAAAATCGAGATCAAGGGCGCAGAC
ACCGACAACTGGGTTGCCTTGGTCCATGACCCAAACTATACGAGTAGCCG
1501 CCCACAAGAACGCTATGGCAGTTGGGTAATCCCACAGGGATCAGGGCCCT
TTAACTTGCCTGTTGGAATTCGTCTGACTAGCCCAACGGGGGAACAGATT
GTGAATGAACAGGCCATCAAGACATTCACTCCTCCGGCCACAGGTGACCC
CAATTTTTACTACATTGACATTGGTGTGCAGTTTAGCCAGAAT**ACTAGT*C***
ACCACCACCACCACCACT**TAACCTAGG**GGTACCGTCGACGGCGCGCCTGCG
1751 GCCGCTTAATTAAGGCCTTGAATCGAGAATTTATACTTAGATAAGTATGT
ACTTACAGGTATATTTCTATGAGATACTGATGTATACATGCATGATAATA
TTTAAACGGTTATTAGTGCCGATTGTCTTGTGCGATAATGACGTTCCTAT
CAAAGCAATACACTTACCACCTATTACATGGGCCAAGAAAATATTTTCGA
ACTTGTTTAGAATATTAGCACAGAGTATATGATGATATCCGTTAGATTAT

2001 GCATGATTCATTCCTACAACTTTTTCGTAGCATAAGGATTAATTACTTGG
ATGCCAATAAAAAAAAAAAACATCGAGAAAATTTCAGCATGCTCAGAAAC
AATTGCAGTGTATCAAAGTAAAAAAAAGATTTTCACTACATGTTCCTTTT
GAAGAAAGAAAATCATGGAACATTAGATTTACAAAAATTTAACCACCGCT
GATTAACGATTAGACCGTTAAGCGCACAACAGGTTATTAGTACAGAGAAA
2251 GCATTCTGTGGTGTTGCCCCGGACTTTCTTTTGCGACATAGGTAAATCGA
ATACCATCATACTATCTTTTCCAATGACTCCCTAAAGAAAGACTCTTCTT
CGATGTTGTATACGTTGGAGCATAGGGCAAGAATTGTGGCTTGAGATCAT
CCTTTTGTTGTTTCCGGGTGTACAATATGGACTTCCTCTTTTCTGGCAAC
CAAACCCATACATCGGGATTCCTATAATACCTTCGTTGGTCTCCCTAACA
2501 TGTAGGTGGCGGAGGGGAGATATACAATAGAACAGATACCAGACAAGACA
TAATGGGCTAAACAAGACTACACCAATTACACTGCCTCATTGATGGTGGT
ACATAACGAACTAATACTGTAGCCCTAGACTTGATAGCCATCATCATATC
GAAGTTTCACTACCCTTTTTCCATTTGCCATCTATTGAAGTAATAATAGG
CGCATGCAACTTCTTTTCTTTTTTTTTCTTTTCTCTCTCCCCCGTTGTTG
2751 TCTCACCATATCCGCAATGACAAAAAAATGATGGAAGACACTAAAGGAAA
AAATTAACGACAAAGACAGCACCAACAGATGTCGTTGTTCCAGAGCTGAT
GAGGGGTATCTCGAAGCACACGAAACTTTTTCCTTCCTTCATTCACGCAC
ACTACTCTCTAATGAGCAACGGTATACGGCCTTCCTTCCAGTTACTTGAA
TTTGAAATAAAAAAAAGTTTGCTGTCTTGCTATCAAGTATAAATAGACCT
3001 GCAATTATTAATCTTTTGTTTCCTCGTCATTGTTCTCGTTCCCTTTCTTC
CTTGTTTCTTTTTCTGCACAATATTTCAAGCTATACCAAGCATACAATCA
AGGAATTCCGGATCCGCCACCATGCCTCAATCCTGGGAAGAACTGGCCGC
TGATAAGCGCGCCCGCCTCGCAAAAACCATCCCTGATGAATGGAAAGTCC
AGACGCTGCCTGCGGAAGACAGCGTTATTGATTTCCCAAAGAAATCGGGG
3251 ATCCTTTCAGAGGCCGAACTGAAGATCACAGAGGCTTCCGCTGCGGATCT
TGTGTCCAAGCTGGCGGCCGGAGAGTTGACCTCGGTGGAAGTTACGCTAG
CATTCTGTAAACGGGCAGCAATCGCCCAGCAGTTAACAAACTGCGCCCAC
GAGTTCTTCCCTGACGCCGCTCTCGCGCAGGCAAGGGAACTCGATGAATA
CTACGCAAAGCACAAGAGACCCGTTGGTCCACTCCATGGCCTCCCCATCT
3501 CTCTCAAAGACCAGCTTCGAGTCAAGGGCTACGAAACATCAATGGGCTAC
ATCTCATGGCTAAACAAGTACGACGAAGGGGACTCGGTTCTGACAACCAT
GCTCCGCAAAGCCGGTGCCGTCTTCTACGTCAAGACCTCTGTCCCGCAGA
CCCTGATGGTCTGCGAGACAGTCAACAACATCATCGGGCGCACCGTCAAC
CCACGCAACAAGAACTGGTCGTGCGGCGGCAGTTCTGGTGGTGAGGGTGC
3751 GATCGTTGGGATTCGTGGTGGCGTCATCGGTGTAGGAACGGATATCGGTG
GCTCGATTCGAGTGCCGGCCGCGTTCAACTTCCTGTACGGTCTAAGGCCG
AGTCATGGGCGGCTGCCGTATGCAAAGATGGCGAACAGCATGGAGGGTCA
GGAGACGGTGCACAGCGTTGTCGGGCCGATTACGCACTCTGTTGAGGACC
TCCGCCTCTTCACCAAATCCGTCCTCGGTCAGGAGCCATGGAAATACGAC
4001 TCCAAGGTCATCCCCATGCCCTGGCGCCAGTCCGAGTCGGACATTATTGC
CTCCAAGATCAAGAACGGCGGGCTCAATATCGGCTACTACAACTTCGACG
GCAATGTCCTTCCACACCCTCCTATCCTGCGCGGCGTGGAAACTACCGTC
GCCGCACTCGCCAAAGCCGGTCACACCGTGACCCCGTGGACGCCATACAA
GCACGATTTCGGCCACGATCTCATCTCCCATATCTACGCGGCTGACGGCA
4251 GCGCCGACGTAATGCGCGATATCAGTGCATCCGGCGAGCCGGCGATTCCA
AATATCAAAGACCTACTGAACCCGAACATCAAAGCTGTTAACATGAACGA
GCTCTGGGACACGCATCTCCAGAAGTGGAATTACCAGATGGAGTACCTTG
AGAAATGGCGGGAGGCTGAAGAAAAGGCCGGGAAGGAACTGGACGCCATC
ATCGCGCCGATTACGCCTACCGCTGCGGTACGGCATGACCAGTTCCGGTA
4501 CTATGGGTATGCCTCTGTGATCAACCTGCTGGATTTCACGAGCGTGGTTG
TTCCGGTTACCTTTGCGGATAAGAACATCGATAAGAAGAATGAGAGTTTC
AAGGCGGTTAGTGAGCTTGATGCCCTCGTGCAGGAAGAGTATGATCCGGA
GGCGTACCATGGGGCACCGGTTGCAGTGCAGGTTATCGGACGGAGACTCA
GTGAAGAGAGGACGTTGGCGATTGCAGAGGAAGTGGGGAAGTTGCTGGGA
4751 AATGTGGTGACTCCATAGCCCGGGGGGGGCTCGATCCCCTCGCGAGTTGG
TTCAGCTGCTGCCTGAGGCTGGACGACCTCGCGGAGTTCTACCGGCAGTG
CAAATCCGTCGGCATCCAGGAAACCAGCAGCGGCTATCCGCGCATCCATG
CCCCCGAACTGCAGGAGTGGGGAGGCACGATGGCCGCTTTGGTCGATCTA
GATTACGTGGAAGAAAGGTAGTAAAAGTAGTAGTATAAGTAGTAAAAAGA
5001 GGTAAAAAGAGAAAACCGGCTACATACTAGAGAAGCACGTACACAAAAAC
TCATAGGCACTTCATCATACGACAGTTTCTTGATGCATTATAATAGTGTA
TTAGATATTTTCAGAAATATGCATAGAACCTCCTCTTGCCTTTACTTTTT
ATACATAGAACATTGGCAGATTTACTTACACTACTTTGTTTCTACGCCAT
TTCTTTTGTTTTCAACACTTAGACAAGTTGTTGAGAACCGGACTACTAAA
5251 AAGCAATGTTCCCACTGAAAATCATGTACCTGCAGGATAATAACCCCCTA
ATTCTGCATCGATCCAGTATGTTTTTTTTTCTCTACTCATTTTTACCTGA
AGATAGAGCTTCTAAAACAAAAAAAATCAGTGATTACATGCATATTGTGT
GTTCTAGTAACCAAAGGAAAGGAACAGATAGATAAAATTCCGAGACTGTC
AAATTAGGTTTTTTTCTTTTTTTTTGGCGGGAGTCAGTGGGCCGAAATAT

5501 GTTCTTGGCCTAGAACTTAATCTGGTTTGATCATGCCAATACTTGCCTGA
GTGCCCGACTTTTTGCCCACCCTCTTGCCTTCTGTCATCCTTCAAAACCC
ACCTGTTTTCCAGCCGTATCTTCGCTCGCATCTACACATACTGTGCCATA
TCTTGTGTGTAGCCGGACGTGACTATGACCAAAAACAAACAAGGAGAACT
GTTCGCCGATTTGTAACACTCCTGCATCCATCCAAGTGGGTATGCGCTAT
5751 GCAATGTTAAGCTAGGTCAGGTCAGACCAGGTCCAAGGACAGCAACTTGA
CTGTATGCAACCTTTACCATCTTTGCACAGAACATACTTGTAGCTAGCTA
GTTACACTTATGGACCGAAAAGGCACCCCACCATGTCTGTCCGGCTTTAG
AGTACGGCCGCAGACCGCTGATTTGCCTTGCCAAGCAGTAGTCACAATGC
ATCGCATGAGCACACGGGCACGGGCACGGGCACAGGAACCATTGGCAAAA
6001 ATACCAGATACACTATACCGACGTATATCAAGCCCAAGTTTAAAATTCCT
AAATTTCCGCGGCTACTTTTCAATTCCCTATAGTGAGTCGTATTAAATTC
GTAATCATGTCATAGCTGTTTCCTGTGTGAAATTGTTATCCGCTCACAAT
TCCACACAACATACGAGCCGGAAGCATAAAGTGTAAAGCCTGGGGTGCCT
AATGAGTGAGCTAACTCACATTAATTGCGTTGCGCTCACTGCCCGCTTTC
6251 CAGTCGGGAAACCTGTCGTGCCAGCTGCATTAATGAATCGGCCAACGCGC
GGGGAGAGGCGGTTTGCGTATTGGGCGCTCTTCCGCTTCCTCGCTCACTG
ACTCGCTGCGCTCGGTCGTTCGGCTGCGGCGAGCGGTATCAGCTCACTCA
AAGGCGGTAATACGGTTATCCACAGAATCAGGGGATAACGCAGGAAAGAA
CATGTGAGCAAAAGGCCAGCAAAAGGCCAGGAACCGTAAAAAGGCCGCGT
6501 TGCTGGCGTTTTTCCATAGGCTCCGCCCCCCTGACGAGCATCACAAAAAT
CGACGCTCAAGTCAGAGGTGGCGAAACCCGACAGGACTATAAAGATACCA
GGCGTTTCCCCCTGGAAGCTCCCTCGTGCGCTCTCCTGTTCCGACCCTGC
CGCTTACCGGATACCTGTCCGCCTTTCTCCCTTCGGGAAGCGTGGCGCTT
TCTCATAGCTCACGCTGTAGGTATCTCAGTTCGGTGTAGGTCGTTCGCTC
6751 CAAGCTGGGCTGTGTGCACGAACCCCCCGTTCAGCCCGACCGCTGCGCCT
TATCCGGTAACTATCGTCTTGAGTCCAACCCGGTAAGACACGACTTATCG
CCACTGGCAGCAGCCACTGGTAACAGGATTAGCAGAGCGAGGTATGTAGG
CGGTGCTACAGAGTTCTTGAAGTGGTGGCCTAACTACGGCTACACTAGAA
GAACAGTATTTGGTATCTGCGCTCTGCTGAAGCCAGTTACCTTCGGAAAA
7001 AGAGTTGGTAGCTCTTGATCCGGCAAACAAACCACCGCTGGTAGCGGTGG
TTTTTTTGTTTGCAAGCAGCAGATTACGCGCAGAAAAAAAGGATCTCAAG
AAGATCCTTTGATCTTTTCTACGGGGTCTGACGCTCAGTGGAACGAAAAC
TCACGTTAAGGGATTTTGGTCATGAGATTATCAAAAAGGATCTTCACCTA
GATCCTTTTAAATTAAAAATGAAGTTTTAAATCAATCTAAAGTATATATG
7251 AGTAAACTTGGTCTGACAGTTACCAATGCTTAATCAGTGAGGCACCTATC
TCAGCGATCTGTCTATTTCGTTCATCCATAGTTGCCTGACTCCCCGTCGT
GTAGATAACTACGATACGGGAGGGCTTACCATCTGGCCCCAGTGCTGCAA
TGATACCGCGAGACCCACGCTCACCGGCTCCAGATTTATCAGCAATAAAC
CAGCCAGCCGGAAGGGCCGAGCGCAGAAGTGGTCCTGCAACTTTATCCGC
7501 CTCCATCCAGTCTATTAATTGTTGCCGGGAAGCTAGAGTAAGTAGTTCGC
CAGTTAATAGTTTGCGCAACGTTGTTGCCATTGCTACAGGCATCGTGGTG
TCACGCTCGTCGTTTGGTATGGCTTCATTCAGCTCCGGTTCCCAACGATC
AAGGCGAGTTACATGATCCCCCATGTTGTGCAAAAAAGCGGTTAGCTCCT
TCGGTCCTCCGATCGTTGTCAGAAGTAAGTTGGCCGCAGTGTTATCACTC
7751 ATGGTTATGGCAGCACTGCATAATTCTCTTACTGTCATGCCATCCGTAAG
ATGCTTTTCTGTGACTGGTGAGTACTCAACCAAGTCATTCTGAGAATAGT
GTATGCGGCGACCGAGTTGCTCTTGCCCGGCGTCAATACGGGATAATACC
GCGCCACATAGCAGAACTTTAAAAGTGCTCATCATTGGAAAACGTTCTTC
GGGGCGAAAACTCTCAAGGATCTTACCGCTGTTGAGATCCAGTTCGATGT
8001 AACCCACTCGTGCACCCAACTGATCTTCAGCATCTTTTACTTTCACCAGC
GTTTCTGGGTGAGCAAAAACAGGAAGGCAAAATGCCGCAAAAAAGGGAAT
AAGGGCGACACGGAAATGTTGAATACTCATACTCTTCCTTTTTCAATATT
ATTGAAGCATTTATCAGGGTTATTGTCTCATGAGCGGATACATATTTGAA
TGTATTTAGAAAAATAAACAAATAGGGGTTCCGCGCACATTTCCCCGAAA
8251 AGTGCCACCTGACGCGCCCTGTAGCGGCGCATTAAGCGCGGCGGGTGTGG
TGGTTACGCGCAGCGTGACCGCTACACTTGCCAGCGCCCTAGCGCCCGCT
CCTTTCGCTTTCTTCCCTTCCTTTCTCGCCACGTTCGCCGGCTTTCCCCG
TCAAGCTCTAAATCGGGGGCTCCCTTTAGGGTTCCGATTTAGTGCTTTAC
GGCACCTCGACCCCAAAAAACTTGATTAGGGTGATGGTTCACGTAGTGGG
8501 CCATCGCCCTGATAGACGGTTTTTCGCCCTTTGACGTTGGAGTCCACGTT
CTTTAATAGTGGACTCTTGTTCCAAACTGGAACAACACTCAACCCTATCT
CGGTCTATTCTTTTGATTTATAAGGGATTTTGCCGATTTCGGCCTATTGG
TTAAAAAATGAGCTGATTTAACAAAAATTTAACGCGAATTTTAACAAAAT
ATTAACGCTTACAATTTCCATTCGCCATTCAGGCTGCGCAACTGTTGGGA
8751 AGGGCGATCGGTGCGGGCCTCTTCGCTATTACGCCAGCTGGCGAAAGGGG
GATGTGCTGCAAGGCGATTAAGTTGGGTAACGCCAGGGTTTTCCCAGTCA
CGACGTTGTAAAACGACGGCCAGTGCCAAGCTCCCGCGGGGATCGACTCA
TAAAATAGTAACCTTCTAATGCGTATCTATTGACTACCAACCATTAGTGT
GGTTGCAGAAGGCGGAATTCTCCCTTCTTCGAATTCAGCTTGCTTTTTCA

9001 TTTTTTATTTTCCATTTTTCAGTTTTTGTTTGTGTCGAATTTAGCCAGTT
GCTTCTCCAAGATGAAAAAAACCCCTGCGCAGTTTCTGTGCTGCAAGATC
CTAATCGACTTTTCCACCCCCCACAAAAGTAAATGTTCTTTTGTTACATT
CGCGTGGGTAGCTAGCTCCCCGAATCTTCAAAGGACTTAGGGACTGCACT
ACATCAGAGTGTGTTCACCTGGTTTGCTGCCTGGTTTGAAAGAAAAGAGC
9251 AGGGAACTCGCGGGTTCCCGGCGAATAATCATGCGATAGTCCTTTGGCCT
TCCAAGTCGCATGTAGAGTAGACAACAGACAGGGAGGGCAGGAAGGATCT
TTCACTGAGATCCTGTATCTTGTTGGGTAAGTCGGATGAAAGGGGAATCG
TATGAGATTGGAGAGGATGCGGAAGAGGTAACGCCTTTTGTTAACTTGTT
TAATTATTATGGGGCAGGCGAGAGGGGGAGGAATGTATGTGTGTGAGGCG
9501 GGCGAGACGGAGCCATCCAGGCCAGGTAGAAATAGAGAAAGCCGAATGTT
AGACAATATGGCAGCGTAGTAGAGTAGGTAGGTAGGCAAGTACTGCTAGC
AAAGAGGAGAAGGGTAAGCTCACTCTTCGCATTCCACACCGTTAGTGTGT
CAGTTTGAACAAAAAAACAATCATCATACCAATTGATGGACTGTGGACTG
GCTTTTGGAACGGCTTTTCGGACTGCGATTATTCGTGAGGAATCAAGGTA
9751 GGAATTTGGTCATATTTACGGACAACAGTGGGTGATTCCCATATGGAGTA
GGAAAACGAGATCATGGTATCCTCAGATATGTTGCGGAATTCTGTTCACC
GCAAAGTTCAGGGTGCTCTGGTGGGTTTCGGTTGGTCTTTGCTTTGCTTC
TCCCTTGTCTTGCATGTTAATAATAGCCTAGCCTGTGAGCCGAAACTTAG
GGTAGGCTTAGTGTTGGAACGTACATATGTATCACGTTGACTTGGTTTAA
10001 CCAGGCGACCTGGTAGCCAGCCATACCCACACACGTTTTTTGTATCTTCA
GTATAGTTGTGAAAAGTGTAGCGGAAATTTGTGGTCCGAGCAACAGCGTC
TTTTTCTAGTAGTGCGGTCGGTTACTTGGTTGACATTGGTATTTGGACTT
TGTTGCTACACCATTCACTACTTGAAGTCGAGTGTGAAGGGTATGATTTC
TAGTGGTGAACACCTTTAGTTACGTAATGTTTTCATTGCTGTTTTACTTG
10251 AGATTTCGATTGAGAAAAAGGTATTTAATAGCTCGAATCAATGTGAGAAC
AGAGAGAAGATGTTCTTCCCTAACTCGAAAGGTATATGAGGCTTGTGTTT
CTTAGGAGAATTATTATTCTTTTGTTATGTTGCGCTTGTAGTTGGAAAAG
GTGAAGAGACAAAAGCTGGAATTGTGAGCGGATAACAAGCTCAACACTTG
AAATTTAGGAAAGAGCAGAATTTGGCAAAAAAAATAAAAAAAAAAATAAAC
10501 ACACATACTCATCGAG
